# Exploring the oncogenic impact of heteroplasmic *de novo MT-ND5* truncating mutations

**DOI:** 10.1016/j.mitoco.2025.03.001

**Published:** 2025-03-26

**Authors:** Yuanyuan Wu, Jiangbin Ye, Zhenglong Gu

**Affiliations:** a Division of Nutritional Sciences, Cornell University, Savage Hall, Ithaca, 14850, NY, USA; b Department of Radiation Oncology, Stanford University School of Medicine, 94305, CA, USA; c Greater Bay Area Institute of Precision Medicine, Fudan University, Nansha District, Guangzhou, 511400, China; d School of Life Sciences, Fudan University, Shanghai, 200433, China

**Keywords:** MT-ND5 variants, mtDNA heteroplasmy, Mitochondrial dysfunction, Oncogenesis

## Abstract

Numerous mitochondrial DNA (mtDNA) variants are associated with cancers, yet the causal link remains inconclusive. Using DddA-derived cytosine base editors, we induced *de novo* truncating mutations in *MT-ND5* in HEK293 cells, establishing heteroplasmy, the coexistence of mutant and wild-type mtDNA. This study aimed to investigate the full molecular etiology following these deleterious mtDNA mutations, particularly in oncogenesis. We found that low to moderate heteroplasmic levels of the mutants were sufficient to impair mitochondrial functions and alter cellular redox status. Cellular adaptation to elevated ROS (Reactive Oxygen Species), energy crisis, and altered redox status was observed across varying heteroplasmy levels. Increased oncogenic potential was confirmed through *in vitro* oncogenesis and *in vivo* xenograft assays. Transcriptomic analysis revealed upregulated migration, invasion, and genome instability pathways, and downregulated ROS scavenging pathways. Our results demonstrate that *MT-ND5* mutations drive cancer progression by increasing cellular ROS and genome instability, and by altering the redox balance and epigenetic landscapes.

## 1. Introduction

A century ago, it was reported that tumor cells exhibited an increased rate of glycolysis despite an adequate oxygen supply, a phenomenon later termed the Warburg effect.^1^ This effect remains a focal point in cancer metabolism research. Initially, Warburg proposed that cancer originated from irreversibly damaged mitochondria, leading to a metabolic shift towards glycolysis.^2^ While this original hypothesis has faced criticism and is considered outdated, the evidence against it does not necessarily contradict Warburg’s hypothesis of impaired mitochondrial function as the origin of cancer. For instance, it has been demonstrated that cancer cells maintain functionally normal mitochondria^3^ and that mitochondrial respiration is essential for cancer cell growth, progression, and metastasis.^4–6^ Moreover, increased aerobic fermentation concurrent with unsuppressed oxidative phosphorylation has been proposed as a more accurate characterization of the Warburg effect.^7–9^ Nonetheless, considering the redundant copies of mitochondria within a single cell, mitochondrial function should not be viewed as a binary system but rather as a spectrum. Thus, the presence of functional mitochondria in established cancer cells, which have undergone extensive clonal expansion and selection,^10–12^ does not necessarily refute the possibility of impaired and suboptimal mitochondrial function in nascent cancer cells.

Indeed, the potential mitochondrial origin of cancer cannot be dismissed, given insights from mutations in nuclear-encoded mitochondrial genes. Key mutations in mitochondrial genes such as *IDH2*,^13,14^ *SDHA*,^15^ and *FH*^16,17^ have been identified as oncogenic. These genetic alterations reprogram cellular metabolism and are implicated in the production of oncometabolites such as 2-hydroxyglutarate (2-HG),^13,18^ succinate,^19^ and fumarate.^15,20^ Dysfunctional mitochondria have been implicated in a wide range of age-related diseases,^21,22^ including neurodegenerative disorders^23^ and cancers.^24^ Their critical role in cellular differentiation^25^ and epigenetic regulation^26,27^ highlights the profound impact of mitochondrial dysfunction on cellular processes.

Mitochondria possess their own genome, comprising 16,569 base pairs, densely encoding 13 coding genes for electron transport chain complexes, and 22 tRNAs and 2 rRNAs necessary for mtDNA translation.^28^ Furthermore, the mitochondrial genome (mtDNA) mutation rate is 10–100x higher than that of the nuclear genome,^29,30^ and the pathogenic mtDNA variants are surprisingly prevalently distributed even among healthy individuals.^31^ Similar to the nuclear-encoded mitochondrial genes introduced earlier, mtDNA variants potentially impact mitochondrial function have been suggested as causal factors in cancer.^32,33^ Despite identification of numerous tumor-specific mtDNA variants through association studies,^34–39^ the driver status of these variants remains to be conclusively determined. To address this, we sought to establish a causal link between mtDNA variants and cancer progression.

However, to determine whether a tumor-specific mtDNA variant is causal in oncogenesis, a meticulously designed study is required.^40,41^ This study must account for differences in the nuclear genome and epigenome, the presence of other complicating mtDNA variants, and the effects of mtDNA copy number and heteroplasmy levels. Only then can the molecular etiology of a specific mtDNA variant be elucidated. Few studies have adhered to these strict criteria, with most employing the transmitochondrial cybrid method to investigate mtDNA variants while controlling for nuclear DNA.^26,42–45^ Despite its importance, this method has limitations. First, the nuclear donor cells in cybrids are often cancerous cell lines, such as the 143 TK-cell line, which are treated with the DNA intercalating agent ethidium bromide (EtBr) for a long period of time to completely eliminate mtDNA, transforming them into rho0 cell lines. Extended EtBr treatment can increase the burden of nuclear DNA mutations, in addition to the original cancerous nuclear genome, obscuring the effects of mtDNA variants. Furthermore, the transmitochondrial cybrid method is restricted to existing mtDNA variants, and the presence of heteroplasmic or unintended mtDNA variants can interfere with the study of the intended variant.

To address these limitations, we proposed investigating the causal link of mtDNA variants to oncogenesis by introducing *de novo* mutations into non-cancerous cell lines and assessing their post-mutation oncogenic potential. Utilizing the recently developed ddA-derived cytosine base editors (DdCBEs), which allow for the induction of *de novo* mtDNA variants,^46^ we induced truncating mutations in *MT-ND5* in the Human Embryonic Kidney (HEK) 293 cell line. This mutation was selected based on previous findings indicating a positive selection of deleterious truncating *MT-ND5* mutations in kidney, colorectal, and thyroid cancers.^39^ *MT-ND5*, the longest mtDNA-encoded gene and a major subunit of mitochondrial Complex I, was selected as it is the most mutated mtDNA-encoded coding gene.^47^

Following the induction of heteroplasmic truncating mutations in *MT-ND5*, we extensively characterized the cellular and mitochondrial responses to these mutations at various heteroplasmy levels. We demonstrated that *MT-ND5* mutations indeed increased oncogenic potential. This study revealed that the induced truncating mutations in *MT-ND5* are advantageous in nascent cancer cells, at least in kidney cells. We reasoned that increased genome instability, elevated ROS levels, and altered epigenetic landscapes are key drivers of the oncogenic potential heightened by the deleterious *MT-ND5* mutations.

## 2. Results

### 2.1. Evaluating MT-ND5 editing efficacy and cellular adaptation to induced heteroplasmy using DdCBEs

**Fig. A.6.**
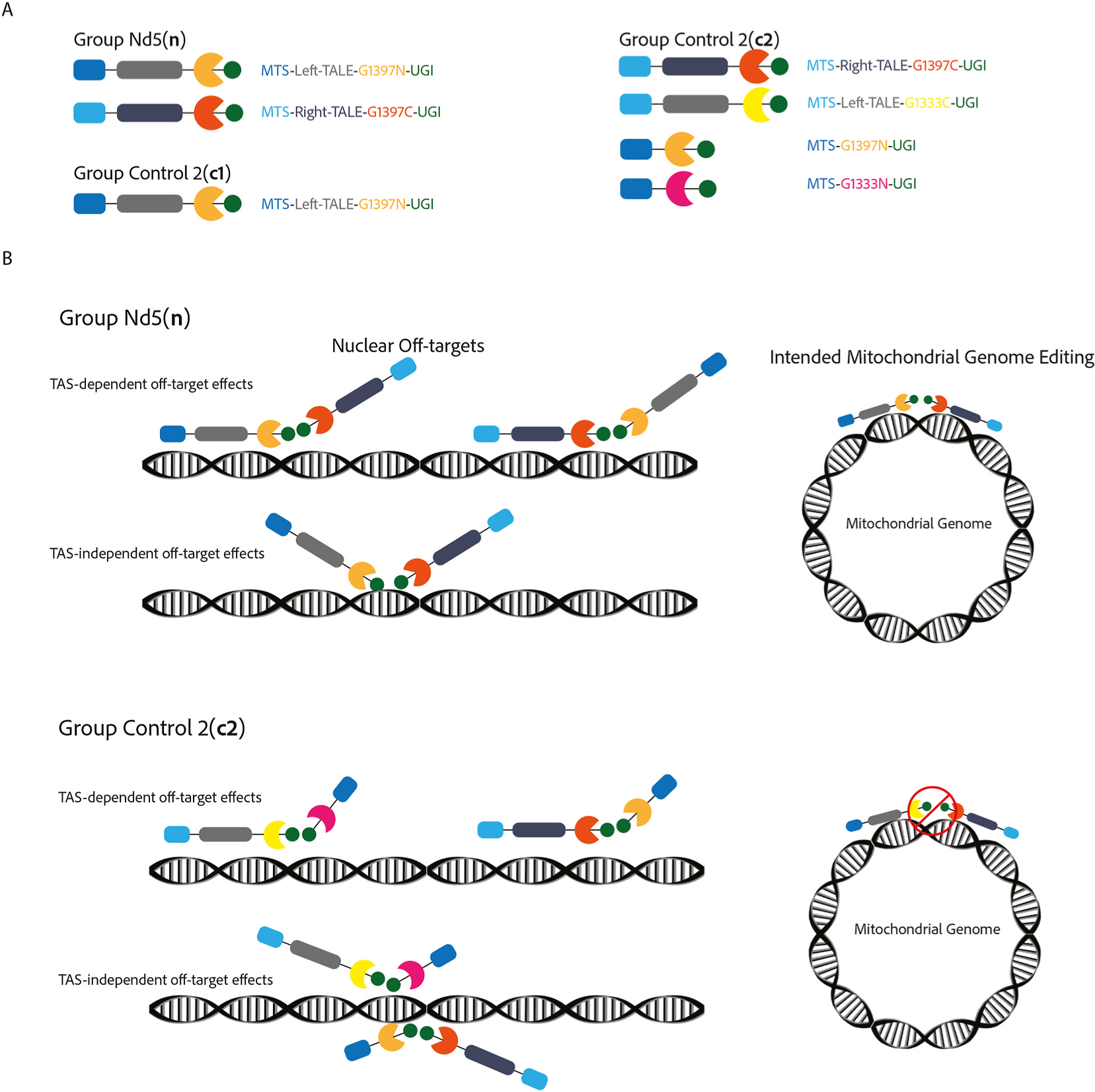
Control Groups Setup for Nuclear Off-targets. A) Setup of control groups for nuclear off-targets. The ‘c1’ group controlled for transfection effect. The ‘c2’ group controlled for nuclear genome off-targets, including both TALE-Array sequence (TAS)-associated off-targets and TAS-independent off-targets. Group ‘n’ was introduced to the *MT-ND5* mutations. B) Demonstration of the c2 group in controlling TAS-dependent and TAS-independent off-targets. Control group c2 was transfected with two confronting DddAtox splits fused with TAS, rendering them non-functional in the mitochondrial genome. Nonetheless, the appropriate paring TAS-free DddAtox splits were also introduced in c2 group, facilitating an accurate representation of TAS-dependent and TAS-independent nuclear off-target effects that might manifest in the treatment group (group n).^48^

First, we aimed to investigate the potential of inducing *de novo* stop gain mutations in *MT-ND5* using bacterial toxin DddA-derived cytosine base editors (DdCBEs).^46^ In canonical DdCBEs, nontoxic and inactive pairing halves of DddA deaminase enzyme were fused with Transcription Activator-Like Effector (TALE) assay sequences (TAS) and transiently transfected for efficient mtDNA editing.^46^ We setup control group c1 to control for merely transfection process, where only an incomplete split component was transfected. Another control group, c2, was setup to control for potential nuclear off-target effects,^48,49^ where the transfected plasmids are confronting and non-functional in mitochondrial genome, but accurately manifest potential nuclear off-target in the *MT-ND5* mutated group n (Figure A.6B). Notably, control groups c1 and c2 are comparable in the majority of the biochemical assays, including two dimensional and anchorage independent growth rate assays, transwell migration and invasion assays, mitochondrial morphology, and energy profile, indicating minimal effects of the rare nuclear off-targets.

**Fig. 1.**
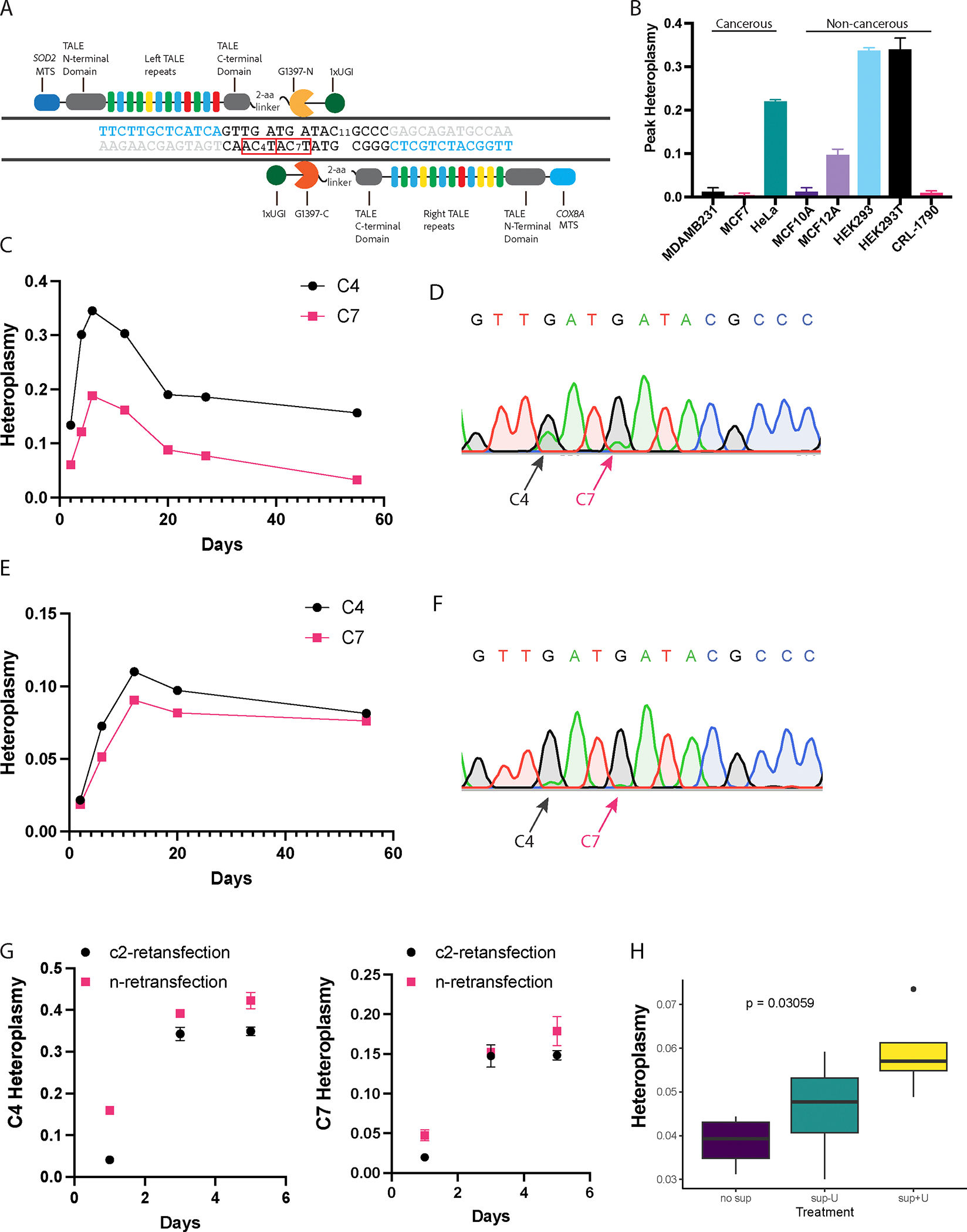
*MT-ND5* editing window, editing efficiency, and cellular tolerance to the induced mutations. A) DdCBE editing window demonstration. Only C4 and C7 were edited within the editing window. B) Peak heteroplasmy across cell lines. C) Temporal changes in heteroplasmy in HEK293. HEK293T cells display a pattern similar to that of HEK293. D) Sanger sequencing trace of editing window on the template strand in HEK293 cells, illustrating a C:T transition in the sense strand. This corresponds to a G:A transition in the displayed template strand. Arrows indicate positions C4 and C7. E) Temporal changes in heteroplasmy in MCF12A. F) Sanger sequencing trace for MCF12A. G) Temporal heteroplasmy change post retransfection for C4 (left panel) and C7 (right panel), for the c2 and n groups. H) Supplements improve heteroplasmy. ‘no sup’, no supplements control group; ‘sup-U’: supplemented with sodium pyruvate, glutamine, and non-essential amino acids; ‘sup + U’: sodium pyruvate, glutamine, nonessential amino acids, and uridine. This assay was conducted in MCF12A cells at early stage heteroplasmy (6 days post-transfection). The Jonckheere-Terpstra test was used to assess trends in heteroplasmy under different treatments. Boxplots represent the median and interquartile ranges. Heteroplasmy level data in B), C), E), and G) represent the mean±sem of technical triplicates.

Upon analyzing the sequence constraints of TALE^50^ and the editing preferences of DdCBEs,^46^ we targeted point mutations at positions MT12806 (C4: the 4th C within the editing window) and MT12809 (C7: the 7th C within the editing window) on the sense strand (Fig. 1A). Cytosine (C) to thymine (T) transitions at these sites were predicted to convert the original mitochondrial tryptophan codon (TGG) to a mitochondrial stop codon (TAA), thereby inducing truncating mutations within *MT-ND5*.

Among all tested cell lines, the highest heteroplasmy level exhibited in HEK293 and HEK293T cell lines (Fig. 1B). Notably, six days post-transfection, both cell lines reached comparable peak heteroplasmy levels of 34 % for C4 and 18 % for C7 (Fig. 1C). The heteroplasmy level specifically refers to the proportion of induced mutant mtDNA. Despite C7 heteroplasmy being uniformly lower, it paralleled C4 in the longitudinal trend over the course of two months (Fig. 1C–E). Consequently, in our analysis, references to heteroplasmy without specifying C4 or C7 pertain to C4 heteroplasmy. In contrast, the MCF10A, CRL-1790, MDA-MB-231, and MCF-7 cell lines showed no detectable heteroplasmy after transfection (Fig. 1B). HeLa and MCF12A cells exhibited lower heteroplasmy than HEK293 cells. We also observed a longitudinal pattern where heteroplasmy peaked and then decayed to a plateau (Fig. 1C–E). These patterns were reproducible across multiple experiments, indicating a robust mutation introduction rate and consistent cellular responses to the mutations. The mechanisms underlying the variance in mtDNA mutation tolerance across cell lines remain unclear. Our data demonstrated that neither the cancerous background of the cell line nor the tissue origin predicted tolerance to the mtDNA variants (Fig. 1B).

To determine whether higher levels of bulk heteroplasmy can be tolerated beyond the observed peak, we conducted a secondary transfection on HEK293 cells. This re-transfection was performed on the previously transfected control group (c2), at a heteroplasmy level of 0 %, and on the treatment group (n), at a stabilized heteroplasmy level of 16 %. As expected, the control group (group c2) manifested the established peak heteroplasmy level of 34 % after retransfection. Notably, the retransfected group n exhibited an increased peak heteroplasmy level of 42 % (Fig. 1G). This new peak heteroplasmy suggests that the retransfection process uniformly converts a proportionate amount of wild-type mtDNA to mutant forms. Specifically, for the C4 editing site, retransfection converted 34 % of the remaining 84 %wild-type mtDNA into mutants. Combined with the pre-existing 16 % mutant mtDNA, this resulted in an approximate new peak heteroplasmy of 42 %. This marks a mutation introduction ceiling of 34 % of wild-type mtDNA in HEK293 cells.

We next investigated the effects of supplements on heteroplasmy levels, anticipating that enhanced nutritional support would mitigate the metabolic deficiency caused by *MT-ND5* mutations. We tested three levels of supplementation, which included either a subset or all of the following nutrients: sodium pyruvate, glutamine, uridine, and non-essential amino acids. These supplements were chosen based on their roles in supporting mitochondrial function and compensating for deficiencies in energy and nucleotide production.^51^ We found a non-random association between the order of the supplementing groups and the heteroplasmy levels (Fig. 1H). This implies that the selection against the mtDNA variants can be partially offset by supplements that bolster mitochondrial function.

### 2.2. Induced heteroplasmic MT-ND5 mutations systematically alter mitochondrial dynamics

We initiated our investigation into the impact of induced mtDNA variants on mitochondrial function at various heteroplasmy levels by assessing Complex I activity. These mtDNA variants were predicted to induce premature termination of transcription in *MT-ND5*, a major subunit of Complex I, potentially affecting its physiological activity. We measured the substrate consumption rate of Complex I at two heteroplasmy levels, a peak of 34 % and a reduced level of 30 %, corresponding to 6 and 12 days post-transfection, respectively.

**Fig. 2.**
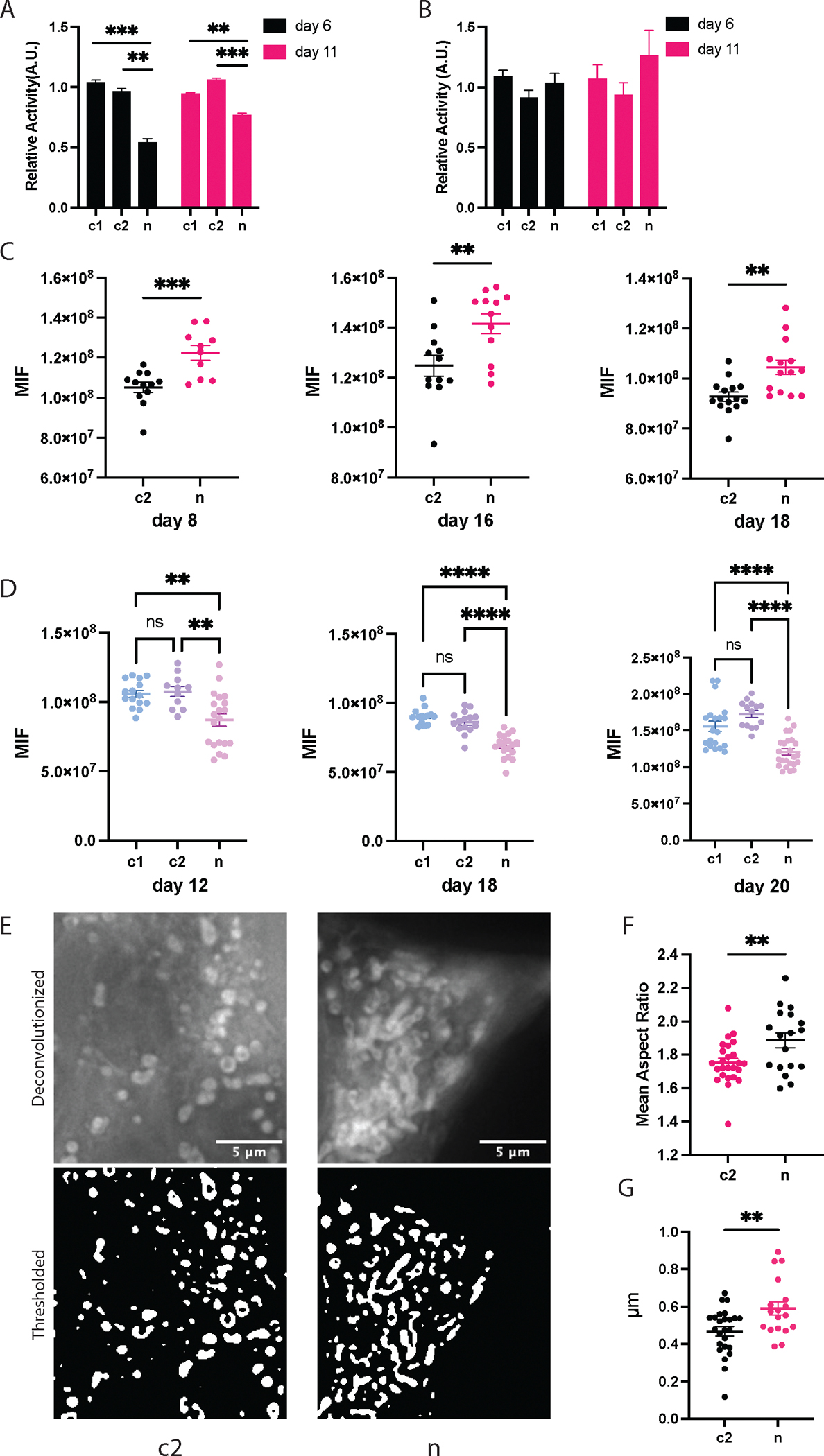
Mitochondrial responses to the induced *MT-ND5* heteroplasmy. A) Relative Complex I activity normalized to citrate synthase activity on day 6 and day 11, at heteroplasmy levels of 34 % and 30 % respectively. B) Relative Complex IV activity normalized to citrate synthase activity. C) MitoSOX median integrated fluorescence (MIF) across treatment on day 8, day 16, and day 18, at the heteroplasmy levels of 33 %, 21 %, and 20 %, respectively. Higher MitoSOX fluorescence indicates a higher mitochondrial ROS level. D) Mitochondrial membrane potential for all treatment groups on day 12, day 18, and day 20, at heteroplasmy level of 30 %, 20 %, and 19 %, respectively. MIF: median integrated fluorescence. E) Representative images for mitochondrial morphology analysis. F) Mean aspect ratio of the identified mitochondria. G) Total branch length per mitochondria. All figures with error bars are mean±sem.

We observed a marked reduction in Complex I activity due to the *MT-ND5* mutations (Fig. 2A). Complex IV activity remained unaltered across all groups (Fig. 2B). Specifically, Complex I activity was approximately 54 % of the control level at 34 % heteroplasmy, improving to 74 % of the control level as the heteroplasmy level decreased to 30 %. This recovery in Complex I activity, with just a 4 % reduction in heteroplasmy over six days, highlights the dynamic and non-linear response of mitochondrial functionality to changes in mtDNA composition.

Next, we investigated the effect of *MT-ND5* mutations on mitochondrial ROS levels. As Complex I is considered a major source of mitochondrial ROS,^52^ we aimed to determine how mitochondrial ROS levels were affected by the dysfunctional *MT-ND5* mutations. The MitoSOX Red dye was utilized to assess mitochondrial superoxide levels. Elevated mitochondrial ROS levels were observed in cells at heteroplasmy levels of 33 % and 20 % (Fig. 2C). Concurrently, we noted a decrease in mitochondrial membrane potential at heteroplasmy levels of 30 %, 20 %, and 19 % (Fig. 2D). Both increased mitochondrial ROS level and reduced membrane potential indicate systematic mitochondrial dysfunction.

Last, we explored the impact of *MT-ND5* mutations on mitochondrial morphology at a near-plateau heteroplasmy level of 20 % (day 18). Cells with *MT-ND5* heteroplasmy exhibited a higher aspect ratio (Fig. 2F) and increased total branch length per mitochondrion (Fig. 2G), suggesting enhanced mitochondrial fusion over fission. This shift towards fusion likely represents an adaptive mitochondrial quality control mechanism aiming to mitigate mutation-induced damage. Fusion potentially helps dilute dysfunctional mutant mtDNA, reflecting cellular efforts to reduce oxidative stress and stabilize membrane potential.^53^ However, this increased fusion activity also hinders complete clearance of mutant mtDNA, stabilizing the deleterious mitochondrial mutations within the mitochondrial population and leading to plateaued heteroplasmy.

### 2.3. MT-ND5 heteroplasmy induces multifaceted cellular responses: Apoptosis, redox status, energy metabolism, and cell cycle arrest

To evaluate the cellular response to the heteroplasmic *MT-ND5* mutations, we first measured the apoptosis rate on days 6 and 16, corresponding to peak heteroplasmy levels of 34 % and 22 %, respectively. We hypothesized that increased apoptosis would selectively eliminate dysfunctional cells, leading to a decrease in heteroplasmy levels. Contrary to our assumption, we observed that all groups exhibited comparably low baseline apoptosis rates (3A). This indicates that the critical biochemical threshold needed to trigger cellular-level dysfunction and apoptosis was not reached at these heteroplasmy level.^54,55^

**Fig. 3.**
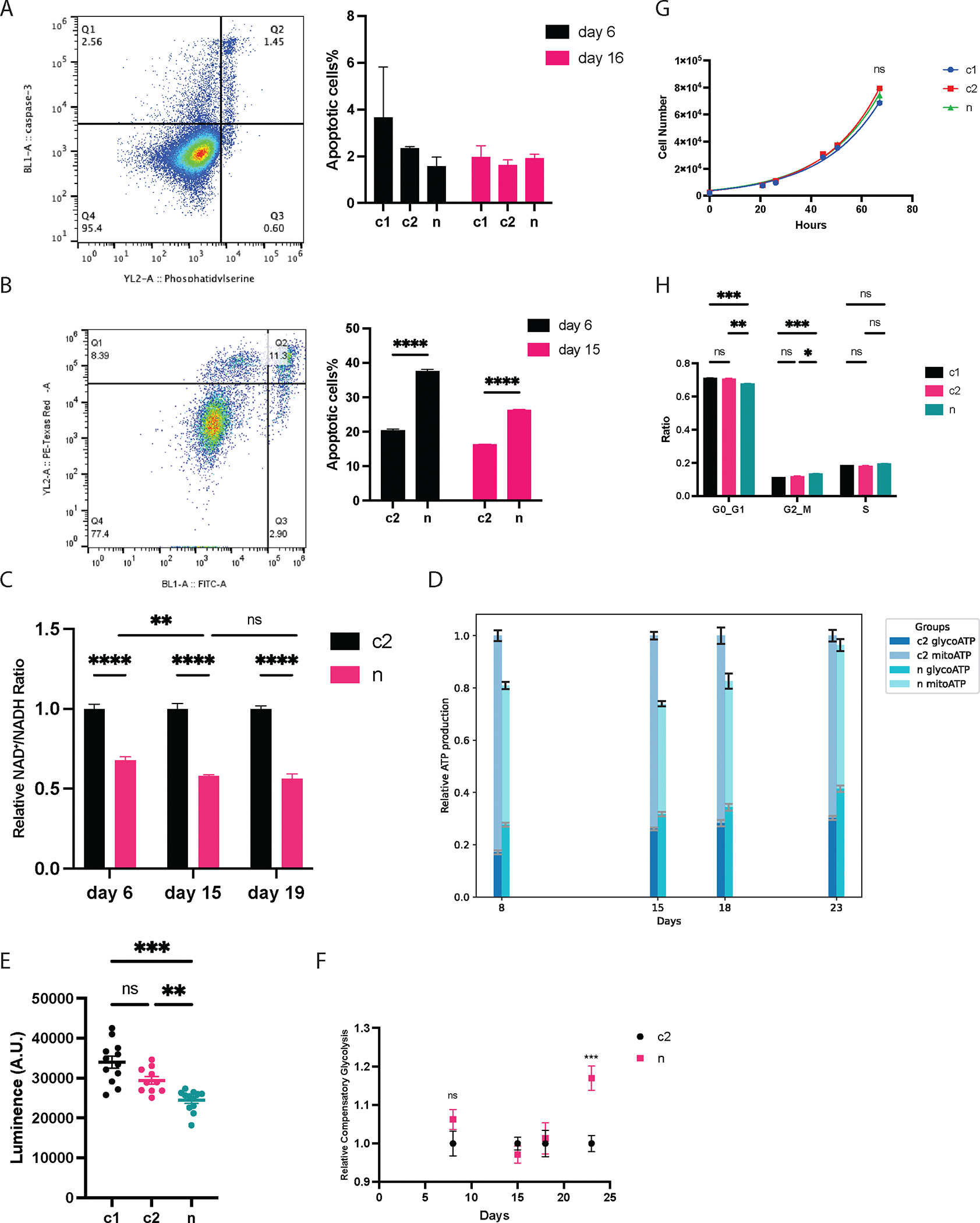
Cellular responses to the *MT-ND5* mutations. A) Left panel: representative flow cytometry dot plot for baseline apoptosis rate. Cells in Q2 were identified as apoptotic cells (phosphatidylserine positive and caspase-3 double positive). Right panel: baseline apoptosis rate for all groups. B) Left panel: representative flow cytometry dot plot for menadione-induced apoptosis rate. Right panel: menadione induced apoptosis rate for all groups. C) NAD^+^/NADH ratio. Each c2/n pair has its ratio normalized to c2. Day 6, day 15, and day 19 represents heteroplasmy levels of 34 %, 30 %, and 20 %, respectively. D) Stacked total energy from glycolysis (glycoATP) and mitochondrial oxidative phosphorylation (mitoATP), as measured and calculated by Seahorse Glycolytic Assay. Each c2/n pair has its value normalized to c2. E) Total ATP estimated from glycolytic assay is validated by CellTiter-Glo 2.0 Cell Viability Assay. F) Compensatory glycolysis, which measures a cell’s maximum capacity for glycolysis, is normalized to the paired c2 for each experiment. G) Representative growth curves for all treatment groups. H) Cell cycle analysis for all groups. All figures with error bars are mean±sem.

Further probing into the cells’ resilience against oxidative stress, we subjected them to menadione, a known inducer of apoptosis through the elevation of cellular ROS levels.^56^ Notably, cells carrying *MT-ND5* heteroplasmy demonstrated heightened susceptibility to ROS-induced stress (Fig. 3B). This sensitivity was more pronounced in cells with 34 % heteroplasmy than in those with 22 %, suggesting that cells with higher heteroplasmy exhibit a more compromised ability to neutralize ROS.

Overall, our results indicate that while *MT-ND5* mutations do not augment baseline apoptosis rate, they render cells more vulnerable to additional oxidative stress. This indicates an exhausted cellular protection against ROS after *MT-ND5* mutations.

Subsequently, we measured the NAD^+^/NADH ratio at a peak heteroplasmy level of 34 %, as well as at 22 % and 20 %, corresponding to growth days 6, 15, and 19, respectively (Fig. 3C). We hypothesized that mutations in *MT-ND5*, a major subunit of mitochondrial Complex I (NADH: ubiquinone oxidoreductase), would decrease the NAD^+^/NADH ratio, as Complex I converts NADH to NAD^+^.^57^

Our results validated the hypothesis, demonstrating a significant reduction in the NAD^+^/NADH ratio in cells across all *MT-ND5* heteroplasmy levels (Fig. 3C). Specifically, from assay days 6–19, as heteroplasmy levels decreased, the NAD^+^/NADH ratio also declined before appearing to stabilize at a lower heteroplasmy. The stabilized NAD^+^/NADH ratio was approximately 56.5 % of the control level (Fig. 3C). This suggests that partial recovery of wild-type mtDNA does not fully mitigate the lasting impact of *MT-ND5* mutations on NAD ^+^ deprivation.

The reduction in NAD^+^/NADH ratio, in combination with increased mitochondrial ROS level and increased susceptibility to induced ROS, indicates an altered cellular redox status after *MT-ND5* mutations.

Next, total cellular ATP was estimated from oxidative phosphorylation (mitoATP) and glycolysis (glycoATP) derived from glycolytic assays (Fig. 3D). This estimation was validated by a luminescent assay on day 18 samples (Fig. 3E), showing comparable relative total ATP ratio to the glycolytic assay. We found that *MT-ND5* mutated cells had a lower overall total ATP level, with the minimal relative total ATP production identified around day 15 (Fig. 3D). Interestingly, on day 23—when heteroplasmy plateaued—the total ATP levels began to normalize, approaching those of the control group (Fig. 3D). We postulate that the energy crisis may select against the *MT-ND5* mutations, hence the stabilization of the heteroplasmy level once the ATP deficit is mitigated.

In parallel, we observed that cells with *MT-ND5* mutations consistently exhibited a 7.59 % increase in glycolysis compared to the control cells (Fig. 3D). While *MT-ND5* mutations resulted in a steady increase in glycolysis, the maximal glycolytic capacity, referred to as ‘compensatory glycolysis’ in the Seahorse Glycolytic Assay, did not exhibit any change until plateaued heteroplasmy (day 23). At this point, it surged by 17 % above the control (Fig. 3F). This significant rise marks the cells’ increasing adaptability to aerobic fermentation at the plateaued heteroplasmy.

We monitored the growth rates of HEK293 cells at heteroplasmy levels of 30 %, 20 %, 19 %, 19 %, and 18 % on days 12, 18, 23, 26, and 30, respectively. Remarkably, no significant changes in growth rates were observed across any of the heteroplasmy levels(Fig. 3G–Table 1). Neither *MT-ND5* heteroplasmy nor supplements altered the cellular growth rate in the MCF12A and HeLa cell lines (Table 1). The sensitivity of our growth rate measurement was validated by assessing TGFB1’s inhibitory effect on MCF12A cells (Table 1), consistent with its known impact on normal mammary epithelial cells.^58–60^ These results confirm that low-to-moderate *MT-ND5* heteroplasmy or supplementation does not impact two-dimensional cell growth rates.

Moreover, we observed notable shifts in the cell cycle distribution of *MT-ND5*-mutated cells (Fig. 3H). Specifically, there was a significant decrease in the proportion of cells in the G0/G1 phase, coupled with an increase in the G2/M phase population (Fig. 3H). The proportion of cells in the S phase remained stable, and this aligns with our findings of unaltered cell growth rate. The exact mechanism underpinning this cell cycle shift remains unclear, yet this observation aligns with a previous report that mitochondrial DNA damages initiate G2/M cell cycle arrest^61^ and another report that G2/M checkpoint pathways are highly associated with mitochondrial genes.^39^

### 2.4. Heteroplasmic MT-ND5 mutations enhance oncogenic phenotypes in vitro and in vivo

**Fig. 4.**
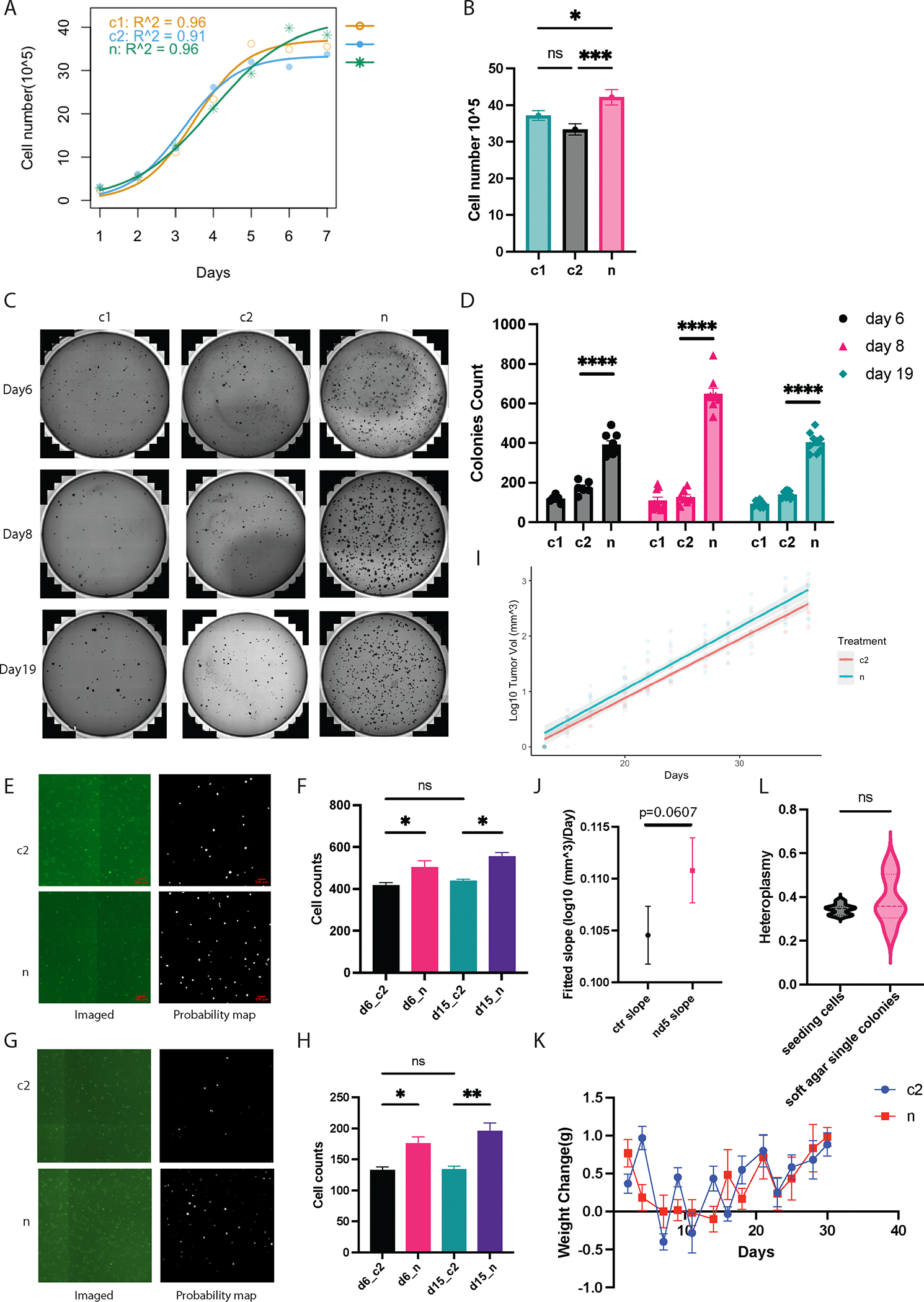
Increased oncogenic phenotypes. A) Logistic model of cell growth after confluence. B) Fitted asymptote from the logistic cell model comparison. C) Representative images of soft agar colonies formed in a single well. D) Summary of soft agar colony counts. E) Representative images of HEK293 transwell migration. F) Quantification of transwell-migrated HEK293 cells. G) Representative images of HEK293 transwell invasion. H) Quantification of transwell invaded HEK293 cells. I) Linear regression of log-wise tumor size to days. Both treatments (c2 and n) are normalized to the first point at which all mice develop tumors. Gray-shaded areas represent error bands indicating the 95 % confidence interval. J) Comparison of fitted slope from linear regression. P-value was derived from the linear mixed effects model for the effect of treatment. K) Mice weight change across days. L) Heteroplasmy of single-cell-derived soft agar colonies does not decay 2 weeks after seeding. All figures with error bars are mean±sem.

To assess the oncogenic potential of *MT-ND5* variants, we evaluated the effects of *MT-ND5* heteroplasmy in HEK293 cells. First, our results indicate that HEK293 cells with *MT-ND5* heteroplasmy exhibit a heightened resistance to contact inhibition, as demonstrated by an elevated fitted asymptote indicative of maximum viable cell count on constrained two-dimensional tissue cultures (Fig. 4A and B).

Subsequent analysis of anchorage-independent growth via the soft agar assay revealed that *MT-ND5* heteroplasmy significantly augments colony formation across heteroplasmy levels (Fig. 4C and D). Notably, the greatest proliferation occurred at a heteroplasmy level of 33 % (day 8). Both peak heteroplasmy levels of 34 % and 20 % facilitated similar, though less pronounced, enhancements in colony formation compared to 33 %.

Additionally, we noted that the average heteroplasmy across all sampled single-cell-derived colonies was consistent with the initial seeding heteroplasmy (Fig. 4L). This indicates that the expansion of colonies in soft agar over a two-week period does not lead to a decline in average heteroplasmy, which contrasts with our observations in standard two-dimensional tissue culture (Fig. 1C–E).

The preservation of heteroplasmy levels within soft agar colonies implies that the selective pressures against mutant mtDNA variants in two-dimensional tissue cultures are either absent or substantially diminished in this three-dimensional, anchorage-independent growth context.

Furthermore, we investigated directed cell motility through transwell migration and transwell invasion assays. Our data demonstrate that both 34 % and 20 % heteroplasmy levels significantly elevate transwell invasion and migration to similar extents (Fig. 4E–H).

We next examined the impact of *MT-ND5* heteroplasmy on tumor growth *in vivo* using a mouse xenograft model. A high number of HEK293 cells (3 × 10^6^ cells/mice) were subcutaneously injected into NOD.Cg-Prkdc^scid^ Il2rg^tm1Wjl^/SzJ (NSG) mice, a strain with severe immune deficiency. Our observations revealed that both the control (c2) and *MT-ND5* heteroplasmic cells (n) exhibited exponential growth following the initial detection of tumors. Notably, there was a trend toward enhanced tumor growth in the presence of *MT-ND5* heteroplasmy (Fig. 4I and J). While the p-value of 0.0607 narrowly exceeds the conventional threshold for statistical significance, there was an estimated exponential daily tumor size increase of 1.45 % compared to the control.

### 2.5. Transcriptomic insights into the oncogenic impacts of the MT-ND5 mutations

**Fig. 5.**
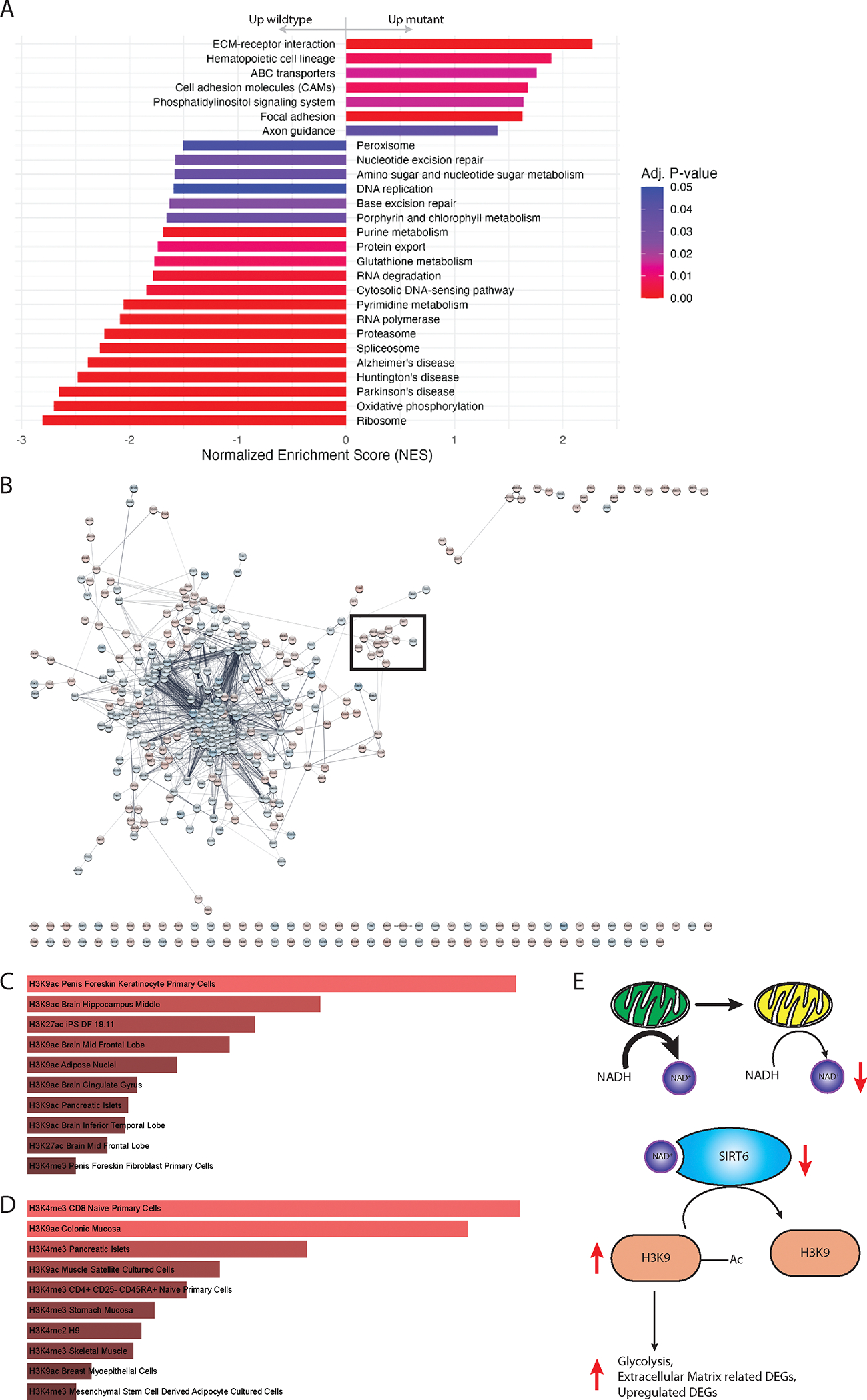
Transcriptomic analysis corroborated the findings of enhanced tumorigenic and metastatic potential. A) Gene set enrichment analysis results. B) Force directed layout display of PPI networks. Boxed cluster is functionally enriched to be migration/invasion related. C) Top 10 enriched histone modifications associated with upregulated DEGs post-*MT-ND5* mutations in HEK293 cells. The Epigenomics Roadmap Histone Modification ChIP-seq gene set was used. Image modified from EnrichR. D) Top 10 enriched pathways for all differentially expressed proteins (DEPs) identified in basal subtype breast cancer against all other breast cancer subtypes. Image modified from Enrichr. E) A model for the activity of SIRT6 and the upregulation of genes regulated by H3K9ac. The upregulation of genes regulated by H3K9ac correlates with decreased activity of SIRT6.

Given the observed oncogenic phenotypes, we conducted a gene set enrichment analysis (GSEA) on the transcriptome data of HEK293 cells to identify pathways responsive to the induced heteroplasmic *MT-ND5* mutations. RNA sequencing data representing three levels of heteroplasmy (34 %, 22 %, and control at 0 %) were utilized for this analysis. The enriched pathways revealed a consistent pro-cancer influence. There was an observed upregulation in pathways linked to cell migration and invasion, including focal adhesion, cell adhesion molecules (CAMs), axon guidance, and ECM-receptor interaction pathways, implying an augmented migrative and invasive capacity (Fig. 5A). The heteroplasmic *MT-ND5* mutations also promote genomic instability. This was supported by the downregulation of the base excision repair pathway, DNA replication pathway and nucleotide excision repair pathway (Fig. 5A). Furthermore, decreased peroxisome pathway and glutathione metabolism pathway (Fig. 5A) indicated an exhausted cellular ability to defend against ROS, agreeing with our observation of increased sensitivity to menadione induced ROS stress (Fig. 3B). Finally, an increase in the phosphatidylinositol signaling system pathway (Fig. 5A) hints at modified cell survival and growth, while a rise in ATP-binding cassette (ABC) transporters suggests a heightened drug resistance potential.

Interestingly, there was an enrichment in multiple pathways associated with neurodegenerative diseases, including Amyotrophic Lateral Sclerosis (ALS), Huntington’s Disease, Alzheimer’s Disease, and Parkinson’s Disease. This aligns with previous findings linking mitochondrial dysfunction to neurodegenerative diseases.^62–65^

Additionally, we plotted protein-protein interaction (PPI) networks to visualize interactions among differentially expressed genes (DEGs). We found that a majority of the affected genes were nuclear mitochondrial genes clustered in the center of the network (Fig. 5B). This finding indicates that the induced *MT-ND5* mutations primarily impacted nuclear-encoded mitochondrial genes. Other closely related identified clusters were functionally enriched for ribosome, Golgi apparatus, and endoplasmic reticulum in the KEGG gene set (Fig. 5B). The proximity of these clusters to the central nuclear mitochondrial gene cluster is in line with expectations, as these organelles are highly reliant on, or functionally related to, mitochondria.^66,67^ It should be noted that the migration/invasion-related cluster, which is functionally enriched for ECM-receptor interaction and PI3K-Akt signaling pathways, was predominantly upregulated (boxed in Fig. 5B). This observation is consistent with the upregulated pathways identified in GSEA (Fig. 5A). However, the upregulated migration/invasion-related cluster is not closely connected to the central, predominantly downregulated clusters. This suggests that the linkage between *MT-ND5* mutation–influenced mitochondrial proteins and the migration/invasion-relevant cluster remains largely unexplored. Alternatively, the upregulation of genes within this migration/invasion-related cluster might be an indirect consequence of the *MT-ND5* mutations.

Hence, we searched for potential indirect effects of the *MT-ND5* heteroplasmy. Our biochemical assays suggested that NAD^+^ (Fig. 3C), as opposed to total ATP (Fig. 3D), served as a critical limiting factor in recovery from the stabilizing *MT-ND5* heteroplasmy. This insight led us to investigate enzymes involved in NAD ^+^ metabolism, with a particular focus on the NAD^+^-dependent class III histone deacetylase (HDAC), especially *SIRT6*. *SIRT6* is notable not only for its role in regulating glycolytic enzymes^68^ but also for its heightened sensitivity to NAD ^+^ levels, evidenced by having the lowest *K*_*m*_ (Michaelis constant) among all sirtuins.^69^

An enrichment analysis of the upregulated DEGs revealed a significant enrichment for genes regulated by H3K9 acetylation (H3K9ac) (Fig. 5C). This result suggests reduced SIRT6 activity, corroborated by our observations of decreased *SIRT6* expression (LogFC: −0.3170546, FDR: 0.02712718) and diminished NAD^+^ concentrations (Fig. 3C).

This finding implies a link between *MT-ND5* mutations and altered epigenetic regulation, potentially mediated through SIRT6 dysfunction. The pronounced enrichment of H3K9ac-regulated genes among upregulated DEGs highlights the impact of *SIRT6* dysregulation, likely exacerbated by reduced NAD^+^ availability. This pathway, connecting mitochondrial dysfunction to epigenetic alterations, offers a plausible explanation for the increased pro-cancer effects observed following *MT-ND5* mutations.

To generalize this model, we extended our research to breast cancer. Notably, the basal subtype of breast cancer exhibits enhanced invasiveness and aggressiveness compared to all other subtypes.^70^ We aimed to investigate whether H3K9ac-regulated pathways are enriched in the basal subtype, potentially accounting for its higher metastatic propensity compared to other subtypes.

Using proteomics data^71^ from the National Cancer Institute’s Clinical Proteomic Tumor Analysis Consortium (CPTAC),^72^ we analyzed differentially expressed proteins (DEPs) in basal subtype breast cancer against other subtypes. Our analysis concentrated on histone modifications and revealed a consistent enrichment for both H3K9ac and H3K4 trimethylation (H3K4me3) (Fig. 5D), aligning with literature that associates these histone modifications with increased metastasis.^73^ This complements our observations that increased H3K9ac histone modifications, potentially resulting from mitochondrial dysfunction and a subsequent decreased SIRT6 activity (Fig. 5E), increased oncogenic potential. Moreover, the involvement of H3K4me3, which requires mitochondrial metabolite alpha-ketoglutarate (aKg) as a cofactor, further demonstrates the significant role of mitochondrial function in metastasis.

## 3. Discussion

In this study, we comprehensively evaluated cellular and mitochondrial responses to induced *MT-ND5* truncating mutations across various heteroplasmy levels. By controlling the nuclear genome, we isolated mtDNA variants to assess their oncogenic effects. Enhanced oncogenic potential was consistently observed across all assays and heteroplasmy levels.

These mtDNA variants induced cell cycle arrest in the G2/M phase and diminished DNA repair pathways, collectively suggesting increased genomic instability. Additionally, our observations of impaired oxidative phosphorylation, elevated mitochondrial ROS, reduced ROS scavenger pathways, and increased menadione-induced apoptosis rates indicate heightened cellular ROS and a compromised defense against ROS. The elevated cellular ROS resulting from impaired oxidative phosphorylation likely contributes to the oncogenic phenotype we observed.^4,45,74^

Furthermore, we monitored the total ATP and NAD^+^/NADH ratio of the cells. As *MT-ND5* heteroplasmy levels decreased, total cellular energy approached normalization near plateaued heteroplasmy. However, the NAD^+^/NADH ratio continued to deteriorate, with the rate of decline slowing and eventually stabilizing as heteroplasmy stabilized within HEK293 cells. This suggests that the NAD^+^/NADH ratio, rather than total ATP levels, may be a critical factor in recovery from *MT-ND5* mutations, possibly explaining the increased demand for NAD ^+^ associated with the Warburg effect.^7,75^ A low NAD^+^/NADH ratio also drives the production of 2-hydroxyglutarate (2-HG),^76–78^ which can inhibit *α*-ketoglutarate (*α*-KG)-dependent dioxygenases, including DNA demethylase TET and JMJD family histone demethylase, causing epigenetic remodeling. Additionally, increased glycolytic capacity was only detected after the total cellular ATP level normalized, indicating that this increase in glycolytic capacity is not driven by energy crisis, but likely by continuing NAD^+^ deprivation. Our findings align with the modern understanding of the Warburg effect, which proposes that the preferential use of aerobic glycolysis in cancer cells, even under normoxic conditions, primarily serves to maintain optimal NAD ^+^ levels rather than to maximize ATP production or biomass synthesis.^7,75^

Multiple studies have indicated that mitochondrial fitness is crucial for the metastatic potential of established cancer cells, with enhanced mitochondrial function correlating with increased metastatic capacity.^4–6^ Collectively, these findings suggest that high mitochondrial fitness is preferentially maintained over dysfunctional mitochondria in established cancer cells. This observation appears contradictory to the numerous tumor-specific mtDNA mutations, including deleterious ones, detected in association tests utilizing sequencing data from established cancer samples.^33,39^ Based on our observations that *MT-ND5* mutations are oncogenic in non-cancerous cells, we propose that the mtDNA mutations observed in cancer tissues may be remnants from the initial stages of oncogenesis, where mtDNA mutations and mitochondrial dysfunction drive the onset of cancer. Within this scenario, deleterious mtDNA variants exhibit a pleiotropic role in cancer: while they contribute to the early stages of cancer formation, they become less adaptive in established cancer cells.

Similarly, current reports suggest that cancer cells maintain a higher NAD^+^/NADH ratio than non-cancerous cells,^79^ in part through increased NAMPT activity to enhance NAD ^+^ production.^80,81^ At first glance, this contrasts with our observation of a lower NAD^+^/NADH ratio and enhanced oncogenic capacity. However, our study focuses on the early responses to *MT-ND5* mutations (within two months) in non-cancerous cells, and the long-term cellular adaptation to chronic NAD^+^ deprivation remains unknown. We speculate that the upregulation of NAMPT and increased glycolysis in established cancers may serve as compensatory mechanisms to restore NAD^+^ levels in response to Complex I deficiency caused by *MT-ND5* mutations. This aligns with our hypothesis that, while mitochondrial dysfunction and NAD^+^ depletion contribute to oncogenesis, they may be actively selected against in fully transformed cancer cells.

Given the lasting impacts of the *MT-ND5* mutations on cellular NAD^+^/NADH ratio, we propose NAD^+^-dependent enzymes, especially the class III HDAC enzyme SIRT6, as promising targets for future research into epigenetic signatures. We propose a model in which decreased NAD ^+^ levels and reduced *SIRT6* expression lead to diminished SIRT6 activity. This decrease in SIRT6 activity results in elevated H3K9ac levels, which subsequently upregulate DEGs associated with enhanced migration and invasion phenotypes, despite the energy crisis stemming from defective mtDNA variants.

Theoretically, deleterious heteroplasmy should be mitigated via mitophagy or apoptosis.^55^ However, our findings of the plateaued heteroplasmy suggest an incapacity of the cells to completely eliminate the deleterious mtDNA variants. This incapacity may be due to recovered total cellular ATP and increased mitochondrial fusion. Moreover, consistent observations of baseline apoptosis rates and unaltered cellular growth rates in two-dimensional cultures suggest that reductions in mtDNA heteroplasmy are attributable to mitochondrial quality control mechanisms, rather than changes at the cellular level. Furthermore, we observed that the single-cell-derived soft agar colonies maintained the peak heteroplasmy as seeding heteroplasmy. It appears that factors unique to a two-dimensional growth matrix, such as direct cell-to-cell interactions or specific metabolic requirements, contribute to the decay of heteroplasmy levels. The cell line–specific tolerance to mtDNA mutation induction implies nuclear control over mtDNA variants.

Based on the oncogenic effects of heteroplasmic *MT-ND5* mutations and the observed plateau in heteroplasmy levels, it remains unclear whether these mutations undergo active selection or persist passively due to impaired mitochondrial quality control. Specifically, the plateaued heteroplasmy could reflect a dynamic equilibrium between mtDNA quality control mechanisms removing mutant mtDNA and a selective advantage promoting its expansion. Alternatively, the persistence of heteroplasmy below a functional threshold may prevent efficient elimination, resulting in passive retention rather than active selection.^55^ Notably, previous studies have reported positive selection for *MT-ND5* truncating mutations in kidney, colorectal, and thyroid cancers, where mutant mtDNA is enriched in tumors relative to surrounding tissues.^39^ In addition, recent discoveries have revealed mitochondrial heterogeneity within cells, with some mitochondria primarily supporting oxidative phosphorylation (OXPHOS) and others facilitating reductive biosynthesis.^82^ This functional diversity may enhance metabolic flexibility, enabling cells to adapt to fluctuating environmental conditions. Therefore, under certain conditions, mutant mtDNA may confer a clonal advantage during tumor evolution, while in other contexts, maintaining heteroplasmy may enhance metabolic flexibility.

Despite the limited low to moderate heteroplasmy levels tested in this study, mitochondrial dysfunction was consistently detected. Our results demonstrate that the mtDNA variants promote aerobic fermentation and are oncogenic, partly aligning with Warburg’s original hypothesis on the mitochondrial origin of cancer. Theoretically, other truncating mutations in *MT-ND5* would contribute similarly. Given the observed incapacity of both the HEK293 and the MCF12A cell lines to completely eliminate mutant mtDNA at lower heteroplasmy levels, and considering the prevalence of heteroplasmic deleterious mtDNA variants even in healthy individuals as reported by,^47^ we suggest a more pivotal role for deleterious mtDNA variants in the initial phases of cancer progression. Specifically, the accumulation of mtDNA mutations initiates mitochondrial dysfunction, which results in increased cellular ROS, altered epigenetics and enhanced genomic instability. These alterations may cumulatively promote the development of nuclear mutator phenotypes, an established modern hypothesis for cancer initiation.^83^

While our study did not directly assess the effects of ROS scavengers or NAD ^+^ supplementation on *MT-ND5* mutation-induced tumorigenesis, the existing literature suggests that both approaches could play a role in mitigating oncogenesis. ROS scavengers have been shown to reduce oxidative stress–induced hyperproliferation^84^ and genomic instability^85,86^ in cancer models. On the other hand, it has also been reported that antioxidant NAC promotes melanoma cell metastasis.^87^ This suggests that the role of ROS scavengers in tumorigenesis may be context- and tumor stage-dependent. Similarly, NAD ^+^ replenishment has been linked to enhanced mitochondrial function,^88,89^ improved cellular repair mechanisms,^90^ and suppression of tumor growth in certain contexts.^91,92^ Future studies are warranted to explore whether these interventions can counteract the oncogenic effects of *MT-ND5* mutations and whether they hold promise as preventive strategies for mitochondrial-driven cancers.

Our investigation was limited to specific mtDNA variants in *MT-ND5* and did not extend to other mitochondrial genes or mutation types, such as missense mutations. The monitoring of heteroplasmy events was restricted to two months, and cellular phenotypes beyond this period were not examined. Considering the chronic and progressive nature of oncogenesis, extending the duration of these studies could provide more definitive evidence of the causal relationship between mtDNA variants and oncogenesis. Further exploration of the epigenomic landscape, especially at the single-cell level where heteroplasmy is not averaged, may further elucidate the mechanisms underlying increased oncogenic potential.

Although our study demonstrated the oncogenic effects of *MT-ND5* mutations in 2D cell culture and in immunocompromised NOD (non-obese diabetic) mice, we proposed that this simplification may underestimate the true impact *in vivo*. First, the nuclear genome of a cell line—HEK293 cell line in this study—is oversimplified compared to the nuclear variability in individuals. Variable nuclear genomes interacting with heteroplasmic *MT-ND5* mutations generate a range of phenotypic variations. Under selective pressures, certain phenotypes may confer a survival advantage, potentially enhancing the oncogenic effects of the *MT-ND5* mutations over time. Second, both 2D growth medium and NOD mice provide levels of glucose in excess of physiological levels and a rich oxygen supply, which do not reflect the nutrient competition and hypoxic microenvironments that cancer cells face *in vivo*. Heteroplasmic *MT-ND5* mutations may confer an adaptive advantage by enabling metabolic plasticity, and this advantage is more pronounced in physiological tumor environments. Additionally, emerging studies suggest that cancer cells can hijack healthy mitochondria from immune cells while dumping dysfunctional mitochondria into T cells, thereby facilitating immune evasion.^93–95^ Cells carrying heteroplasmic *MT-ND5* mutations may exploit similar mechanisms to escape immune surveillance, which was not captured in our immune-deficient model. Future studies utilizing non-cancerous murine cell lines and immunocompetent mice will be essential to elucidate the long-term oncogenic effects of *MT-ND5* mutations in a more physiologically relevant context.

In conclusion, our research establishes a causal link between specific deleterious mtDNA variants and oncogenesis. We demonstrated that even moderate levels of these mutations result in impaired mitochondrial function, increased aerobic fermentation (the Warburg effect), and an enhanced oncogenesis phenotype. Our characterization of the cellular and mitochondrial responses to the heteroplasmic mtDNA variants advances the understanding of fundamental mitochondrial biology. Our findings not only elucidate the pivotal role of mtDNA mutations in oncogenesis but also pave the way for cancer prevention and early detection, as well as future investigations into targeted therapies that may mitigate the oncogenic potential of the common deleterious mtDNA mutations.

## 4. Methods

### 4.1. Cell culture

The HEK293, HEK293T, HeLa, MDA-MB-231, and MCF-7 cell lines were cultured in DMEM (Gibco, #11965118) supplemented with 10 % FBS (Avantor Seradigm, #1300–500) and 100 U/ml Penicillin-Streptomycin (Gibco, #15140122). The CRL-1790 line was maintained in MEM (Gibco, #11095080) with identical supplementation. The MCF10A and MCF12A lines were cultured in DMEM/F12 (Gibco, #11320033) supplemented with 10 μg/ml recombinant insulin (Sigma, #45-I0516–5 ML-EA), 20 ng/ml EGF (Peprotech, #AF-100–15), 100 ng/ml cholera toxin (Sigma, #45-C8052–1 MG), 500 ng/ml hydrocortisone (Sigma, #45-H0888–1G-EA), 5 % Horse serum (Life Technologies, #26050088), and 100 U/ml Penicillin-Streptomycin. All cell cultures were maintained in a humidified incubator at 37 °C with 5 % *CO*_2_. Regular screening for mycoplasma was conducted using the LOOKOUT MYCOPLASMA PCR DETECTION KIT (Sigma, #45-MP0035–1 KT-EA).

For experiments examining supplement effects, reagents were added to achieve final concentrations of 1 mM sodium pyruvate (Gibco, #11360070), 1x non-essential amino acids (Gibco, #11140050), 1x GlutaMAX (Gibco, #35050061), with or without 50 μg/ml uridine. When examining the effects of TGFB1 on MCF12A cells, 20 ng/ml of TGFB1 (Gibco, #PHG9204) was supplemented.

### 4.2. Plasmid construction

Polymerase chain reaction (PCR) was performed using Phusion Hot Start II DNA Polymerase (Thermo Scientific, #F549L). DdCBE splits were synthesized as gene blocks and codon optimized for human expression (Genscript). The mitoTALE sequences were engineered employing the TALE toolbox (Addgene kit # 1000000019), following the detailed protocol.^96^ Both DdCBE splits alone and those coupled with mitoTALE were cloned into the pCMV vector backbone, ensuring an overlap of 20–25 nucleotides (nts) via Gibson Assembly (New England BioLabs, Catalog #E2621L). Subsequent to the Gibson Assembly, the products were column-purified prior to their introduction into Stbl3 Chemically Competent *E. coli* (Thermo Scientific, Catalog #C737303) to preclude any buffer incompatibilities. The plasmids destined for mammalian transfection were purified employing ZymoPURE II Plasmid Midiprep Kits (Zymo Research) following the manufacturer’s protocol. The DNA sequences of all the plasmids utilized in this study are listed in the supplementary materials.

### 4.3. Mitochondrial DNA editing via transient plasmid transfection

Cells were enzymatically dissociated for transfection using TrypLE Express Enzyme (Gibco, #12605028) one day before the procedure, and their numbers were subsequently quantified with a TC20 Automated Cell Counter (Bio-Rad). We then seeded 3 × 10^5^ cells per well into 6-well plates (VWR International, #10062–892). The following day, for transfection, 4 μg of plasmids was introduced into the cells with Lipofectamine 2000 (Invitrogen, #11668027), in line with the manufacturer’s protocols. The *MT-ND5* heteroplasmic group (n) received two plasmids, 1L-1397N and 1R-1397C, each at 2 μg, equipped with the necessary components for precise mitochondrial DNA (mtDNA) editing at predetermined sites. Control group 1 (c1) was transfected with a single plasmid, 1L-1397N, at 4 μg, serving as a baseline to evaluate any inherent effects resulting from the transfection process. Control group 2 (c2) was transfected with four plasmids, each at 1 μg, to assess TALE array sequence (TAS)-dependent or independent off-target effects on the nuclear genome (Figure A.6B). This group included TAS-containing plasmids 1R-1397C and 1L-1333C, alongside DddA split-only plasmids 1397N and 1333N. Although the TAS-paired plasmids were predicted to be inactive in mitochondrial editing due to mismatches in the deaminase splits, the collective use of these four plasmids was engineered to emulate the nuclear off-target effects observed in the *MT-ND5* heteroplasmic group (n), thus allowing for a controlled comparison.

### 4.4. MT-ND5 heteroplasmy quantification

The DNA from cultured cells was isolated using the Wizard Genomic DNA Purification Kit (Promega) or by direct boiling with RNase A and Proteinase K. Following purification, the DNA was amplified with Phusion Hot Start II DNA Polymerase (Thermo Scientific, #F549L) using the following primers for PCR: A19 (5’-TTACCGAGAAAGCTCACAAGAA-3’) and B22 (5’-CGTGAAGGTAGCGGAT GATT-3’). The PCR cycling conditions were set as follows: an initial hold at 98 °C for 30 s, followed by 35 cycles of denaturation at 98 °C for 10 s, annealing at 62 °C for 30 s, and extension at 72 °C for 1 min and 45 s, with a final extension step at 72 °C for 10 min. The resulting PCR products were then electrophoresed on a 0.8 % agarose gel in TBE buffer to confirm the presence of a unique band.

Post-electrophoresis, PCR products were purified using either the Monarch PCR & DNA Cleanup Kit (NEB, #T1030L) or the 96 DNA Clean & Concentrator-5 (Zymo, #11–306A) for high-throughput applications. The purified DNA concentrations were determined spectrophotometrically using a NanoDrop instrument, after which the samples underwent Sanger sequencing, using A19 for primer. Relative fluorescence intensities of the wildtype and mutant nucleotides were subsequently read with SnapGene software. Heteroplasmy levels were calculated as follows:

$$Heteroplasmy\;level\;\%=\frac{\;\text{mutant}\;}{\;\text{mutant}\;+\;\text{wildtype}\;}\times 100\%$$

### 4.5. Glycolytic assays

Mito-stress and glycolytic assays were conducted on the Seahorse XF24 (Agilent) according to the manufacturer’s guidelines. Assay plates were pre-treated with poly-L-serine (Sigma), and 4 × 10^4^ cells were seeded 24–48 h prior to the assay. Seahorse XF DMEM, pH 7.4 (Agilent, #103575–100), was used as the assay medium. For the glycolytic assay, the medium composition included 10 mM glucose, 1 mM sodium pyruvate, and 2 mM glutamine, with drugs added at final concentrations of 0.5 μM Antimycin, 0.5 μM Rotenone, and 50 mM 2-DG. Following the Seahorse assay, cells were stained with Hoechst33342 (Thermo Fisher, #H3570) and counted using the Celigo Image Cytometer (Nexcelom). Both OCR and ECAR values were normalized to the total cell count.

Estimation of glycolytic ATP (glycoATP) and mitochondrial ATP (mitoATP) was performed using the glycolytic assay readout. Detailed calculation methods are provided in the supplementary materials.

### 4.6. Cell growth rate quantification

Total cell count with at least triplicates were counted for at least 4 timepoints to monitor the cell growth rate. Specifically, cells were seeded at 1000 cells/well in a 96 well plate, with at least 15 wells seeded for each treatment. This allowed longitudinal cell number tracking with triplicates for a maximum of 5 timepoints. After overnight seeding in standard cell growth condition, the total cell number was counted with Celigo image cytometer and Hoechst 33342 dye.

To assess the impact of *MT-ND5* heteroplasmy on cell growth, a linear regression model was applied using the logarithm of cell counts as the response variable. The model was structured to include interactions between time (measured in hours) and treatment, enabling the investigation of how treatment effects evolve over time. In the case of MCF12A cells, where treatments involved combinations of *MT-ND5* and TGFB1, a linear regression model was formulated with interaction terms for both *MT-ND5* and TGFB1. These terms interact with Time, capturing both the main effects of time and of treatments as well as their interaction effects. Specifically,

$${log}_{10}(\text{cell}\;\text{count})=\beta_{0}+\beta_{1}(\text{treatment})+\beta_{2}(\text{time})\;\;+\beta_{3}(\text{treatment}\times \text{time})+\epsilon$$.

The fitting was conducted in R. The statistical significance of the treatment was determined from the model’s summary.

### 4.7. Total cellular ATP quantification

To validate the total cellular ATP estimated from glycolytic assays, total cellular ATP was measured using the CellTiter-Glo 2.0 Cell Viability Assay (Promega, #G9241), with the procedure carried out according to the manufacturer’s instructions. Luminescence readings were taken with a SpectraMax M3 microplate reader, utilizing a white 96-well plate. Cells were seeded at a density of 2 × 10^4^ cells per well, 24 h prior to the assay, and at least five wells were designated for normalization purposes. For normalization, the total number of cells was accurately quantified using a Celigo cell cytometer, with Hoechst dye employed to facilitate the cell counting process.

### 4.8. NAD^+^/NADH ratio quantification

The NAD^+^/NADH ratio was determined using the NAD/NADH-Glo Assay (Promega, #G9071), adhering to the manufacturer’s protocols. Specifically, 2 × 10^4^ cells were plated 24 h before the assay. Luminescence was longitudinally measured with a SpectraMax M3 microplate reader in a white 96-well plate. The slope of the fitted curve, representing luminescence as a function of time, was utilized to estimate the quantities of NAD^+^ and NADH, which were then used to calculate the NAD^+^/NADH ratio. The standard error of the NAD^+^/NADH ratio was derived from the standard errors of the fitted slopes for NAD^+^ and NADH, using the delta method for error propagation. To evaluate the differences in the NAD^+^/NADH ratio among the groups, based on the obtained ratios and their respective standard errors, an ordinary one-way ANOVA was employed. The post hoc analysis for multiple comparisons was corrected using the two-stage step-up method of Benjamini, Krieger, and Yekutieli.

### 4.9. Apoptosis assay

We used the Dual Apoptosis Assay Kit with NucView 488 Caspase-3 Substrate & Sulforhodamine 101-Annexin V (Biotium, #30030) according to the manufacturer’s protocol on an Attune NxT flow cytometer (Thermo Fisher). Cells were seeded at 2 × 10^5^/well in a 24-well plate, with or without 10 μM menadione for 12 h. Data analysis was conducted in FlowJo.

### 4.10. Transwell invasion/transwell migration assays

For the transwell invasion assay, a 24-well plate with an 8.0-μm Matrigel invasion chamber (Corning, #354480) was employed. The transwell migration assay utilized 8.0-μm cell culture inserts (Corning #353097). Both assays were conducted in line with the manufacturer’s guidelines. Specifically, 2.5 × 10^4^ cells were seeded per well for HEK293. Normal complete cell culture medium was used as a chemoattractant, with 10 % FBS supplementation.

After incubating for 16–24 h in a cell incubator, cells were either stained with CellTracker Green CMFDA Dye (Life technologies, #C2925) for live imaging. Live imaging was conducted instead of fixation with crystal violet to prevent cell detachment, as HEK293 cells exhibit loose attachment. Imaging was executed using a Celigo cell cytometer. All captured images were stitched together and exported at a resolution of 3 μm/pixel. Analysis in ImageJ/Fiji quantified the total number of invaded or migrated cells. The ‘Trainable Weka’ plugin was employed for accurate cell identification against the background pores in whole-mount images. Representative cells were manually labeled to guide Trainable Weka’s supervised learning, producing a probability map. This map was then thresholded for cell counting. ImageJ macros were used for both thresholding and quantification, with the corresponding scripts provided in the supplementary materials.

### 4.11. Mitochondrial membrane potential and mitochondrial oxidative stress

To assess mitochondrial ROS stress and mitochondrial membrane potential, MitoSOXRed (Thermo Fisher, #M36008) at 5 μM and TMRM (Thermo Fisher, #T668) at 20 nM were employed, following the manufacturer’s guidelines for each, respectively. Cells were seeded in a 35 mm glass bottom plate (Mat Trek) and were allowed to adhere for 24 h prior to imaging. Live imaging was performed on the LionHeart imaging station (Agilent) with a 40x objective at 37 °C in phenol red–free DMEM growth medium. Along with the mitochondrial dyes, Hoechst33342 was introduced. This dye was used as the primary mask and was extended by 10 μm to serve as the secondary mask, allowing for the determination of the integral red fluorescence intensity of each cell. Analysis of this fluorescence was carried out using the BioTek Gen5 software (Agilent). The median fluorescence from each field was documented, and at least 10 distinct, non-overlapping fields were examined for each treatment. Statistical tests were performed with GraphPad Prism 9.

### 4.12. Cell cycle analysis

Cells were harvest and counted with a TC20 Automated Cell Counter (Bio-Rad) to ensure >95 % cell viability. Then the cells were washed and fixed with ice-cold methanal. Post fixation, the cells were washed twice again and stained with a solution containing 50 μg/mL propidium iodide (PI) and 100 μg/mL RNase A, followed by incubation at 37 °C for 30 min in the dark. Stained cells were then analyzed on an Attune NxT flow cytometer (Thermo Fisher).

### 4.13. Soft agar assay

Soft agar assay was conducted as outlined in a previously reported method,^97^ with minor adjustments. Specifically, noble agar solutions of 1 % and 0.6 % were prepared in deionized water and subsequently autoclaved. A 2x concentration of DMEM medium was formulated from powder and sterilized using a 0.2 μm filter. On the assay day, a fresh 0.5 % bottom agar was made by mixing equal parts of the 1 % agar and 2x complete growth medium, which had twice the usual supplements. This was allowed to set at room temperature in the cell hood for at least 2 h. Log-phase cells were harvested and dissociated with Tryple, followed by passing through a 40 μm strainer twice through a 25 gauge needle to ensure a single-cell solution. 2000 cells/well were incorporated into a mixture of prewarmed 2x complete growth medium and 0.6 % agar, producing a 0.3 % top agar. This top layer was then left to solidify for a minimum of 1 h at room temperature in the hood. Subsequently, the plates were placed in the cell incubator at 37 °C with 5 % *CO*_2_ for 12 days, with 100 μL of complete growth medium added every 3 days to prevent drying. At the end of the incubation, 200 μL of 1 mg/ml nitroblue tetrazolium chloride (NBT) solution was added to each plate, and they were incubated overnight. Imaging was performed using Celigo image cytometer. Statistical tests were performed with GraphPad Prism 9.

### 4.14. Mitochondrial morphology

Cells were stained with 20 nM TMRM and Hoechst33342 as per the manufacturer’s instructions. Live imaging of stained cells was performed in phenol red–free complete medium using a LionHeart imaging station (Agilent) at 37 °C with a 100x objective. We captured Z-stacks of a minimum of 25 slices, each 100 nm deep. From each imaging field, subsets of Z-stacks (273 pixels × 293 pixels × 25 slices) containing mitochondria from a single cell were chosen for analysis in ImageJ, following previously established methods.^98^ These selected Z-stacks were considered as the ‘cell’ for later analysis.

The cell stacks underwent deconvolution using DeconvolutionLab2 in ImageJ, utilizing a theoretical Point-Spread Function (PSF) produced by the ‘PSF generator’ plugin. The Richardson-Lucy TV algorithm was applied, with a regularization value of 0.0001 and 30 iterations, consistent with the established method.^98^ Post-deconvolution, the image edges were cropped to mitigate edge effects during deconvolution. The 3D stacks were then converted to 2D images via maximal projection. These 2D images were subsequently analyzed using the ‘Mitochondrial Analyzer’ plugin to assess cell morphology metrics. The analysis parameters included the ‘weighted mean’ threshold method, a block size of 1.45 μm, and a C-value of 8.

### 4.15. Spectrometric assessment of the enzymatic activities of mitochondrial complexes

Mitochondrial complex activity was assessed with modifications to previously reported protocols.^99,100^ Mitochondria were isolated from an equal number of cultured cells across all samples using the Qproteome Mitochondria Isolation Kit (Qiagen, #37612), adhering to the manufacturer’s standard procedure. The isolated mitochondria were resuspended in a hypotonic buffer (10 mM Tris-HCl), snap-frozen in liquid nitrogen, and then stored at −80 °C until spectrometric analysis could be performed. A fraction of the resuspended mitochondria was assayed for total protein content using the BioRad Protein Assay Kit, following the manufacturer’s guidelines. On the day of the spectrometric assessment, the mitochondrial suspensions underwent three freeze-thaw cycles in the hypotonic buffer to ensure membrane disruption and release of the mitochondrial enzymes.

The spectrometric readings were taken using the SpectraMax M3 (Molecular Devices) microplate reader, set to 37 °C, in black-bottom 96-well plates. All required buffers and reagents were prepared in alignment with the established protocols.^99^ Longitudinal spectrometric measurements were carried out to evaluate the activities of Complex I (NADH:CoQ oxidoreductase), Complex IV (cytochrome *c* oxidase), and the internal control enzyme citrate synthase, as detailed in the modified method.^100^

Enzymatic activities and their standard errors were calculated from the slopes of the longitudinal readouts. Both Complex I and Complex IV activities were normalized against citrate synthase activity to account for variations in mitochondrial content. The standard errors of the normalized activities for Complexes I and IV were computed using the delta method, which considers the propagation of errors through calculations involving multiple variables. Statistical analysis of group differences in complex activities was performed using Brown-Forsythe and Welch ANOVA tests, with post hoc multiple comparisons corrected by Dunnett’s T3 method to control for type I errors.

### 4.16. RNA isolation, library preparation, sequencing, and analysis

Total RNA was isolated from cultured cells using TRIzol Reagent following the manufacturer’s instructions. The RNA was spectrophotometrically quantified using Nanodrop. Prior to RNA-sequencing, RNA integrity was assessed using the Fragment Analyzer (Agilent), ensuring all samples exhibited an RNA quality number exceeding 9.0. Libraries were prepared using the NEBNext Ultra II [Directional] RNA PolyA enrichment kit as per the manufacturer’s guidelines. Subsequent quantification of each library was performed using the Qubit (dsDNA HS kit; Thermo Fisher), and size distribution was ascertained with a Fragment Analyzer (Agilent) before pooling. Sequencing was conducted on a NovaSeq platform, producing a minimum of 20M 150 nucleotide paired-end reads for each library. Reads underwent trimming to remove low-quality and adaptor sequences with the aid of TrimGalore v0.6.0,^101^ a tool that incorporates both cutadapt^102^ and fastQC.^103^ These reads were then aligned to the reference transcriptome (Ensembl GRCh38) utilizing STAR v2.7.0.^104^

The EdgeR package^105^ facilitated the filtering and normalization of raw read counts. Analyses were subsequently conducted using a linear model tailored to account for variations in heteroplasmy levels, with adjustments corresponding to each 34 % change in heteroplasmy. This specific threshold was determined based on the highest level of heteroplasmy observed in the HEK293 cell line. Following this, dispersion values were computed. A likelihood ratio test was then applied to identify genes that exhibited significant correlations with the heteroplasmy levels.

### 4.17. Gene set enrichment analysis (GSEA)

Preranked GSEA was conducted with HEK293 RNA-seq data, ranking with signed log(P value) generated from RNA seq analysis with EdgeR, as previously described. The ‘fgsea’ library was employed on the KEGG pathways gene set. We adopted an adjusted p-value threshold of < 0.05 to determine the significance of the enriched pathways.

Gene ontology was also conducted. Briefly, for the HEK293 RNA-seq data, DEGs were identified as described in the RNA-seq analysis subsection, with EdgeR and a GLM model. To capture nuanced change following the *MT-ND5*, a log2(Fold change) of 0.2 was chosen to cutoff for the upregulated DEGs, combining a FDR<0.05. The upregulated DEGs were then pasted into the online tool Enrichr^106–108^ to identify enriched pathways in the Epigenomics Roadmap HM ChIP-seq gene set. Similarly, public breast cancer proteome data^71^ from the Clinical Proteomic Tumor Analysis Consortium (CPTAC)^72^ were used. Differentially expressed proteins (DEPs) of the basal subtype breast cancer against all other breast cancer subtypes were identified with cutoffs of 5 and 2 for log(FDR) and —log2(Fold Change)—, respectively.

### 4.18. Protein-protein interaction (PPI)

The protein-protein interaction map was constructed using Cytoscape (version 3.10.1).^109^ Differentially expressed genes (DEGs) were identified with the EdgeR package as described above, requiring an FDR of less than 0.05 for inclusion in the PPI analysis. These DEGs were subsequently analyzed using the STRING database to establish the interaction network. The network was organized into clusters by applying the Markov Cluster Algorithm (MCL), which is integrated within the STRING tool. To visualize the resulting subclusters, a force-directed layout was implemented in Cytoscape. The node filling color used a continuous scale, with red indicating upregulated DEGs and blue indicating downregulated DEGs.

### 4.19. Mice xenograft

NOD.Cg-Prkdc^scid^ Il2rg^tm1Wjl^/SzJ (NSG) immunocompromised mice (strain #005557) were obtained from Jackson Laboratories and subsequently bred in-house at Cornell University, Ithaca, as part of the institution’s animal breeding program. The transplantation and subsequent tumor monitoring were conducted by the Progressive Assessment of Therapeutics (PATh) Patient-Derived Xenograft (PDX) Facility at Cornell University. This study utilized twelve young adult female NSG mice, which were allocated equally into control and treatment groups (n=6 per group). Prior to transplantation, the cells were cultured in an antibiotic-free growth medium for two weeks and assessed for mycoplasma contamination. After harvest, the cells were suspended in cold phosphate-buffered saline (PBS) and maintained on ice before being combined with an equal volume of Matrigel. A 200 μL mixture containing 3 × 10^6^ cells was administrated subcutaneously into the mice. Tumor weight and size were monitored bi-weekly or tri-weekly using calipers, with the tumor volume calculated using the following formula: *Volume* = (*Length* × *Width*^2^) × 0.5.

For statistical analysis, we employed a linear mixed-effects model with restricted maximum likelihood (REML) estimation using the gathered longitudinal tumor volume data. The model specified log-transformed tumor volume (*log*_10_[*Volume*]) as the dependent variable and included fixed effects for treatment group, time (in days), and their interaction. We incorporated a random intercept to account for the repeated measures and intrinsic variability among the individual mice. The regression was conducted in R with the ‘lme4’ and ‘lmerTest’ packages.

## Supplementary Material

Figure Supplement 1

Supplement_2_plasmid_sequence

## Figures and Tables

**Table 1:** Effect of treatment on growth rate across multiple cell lines. P-value is derived from a log-linear mixed-effects model assessing the impact of treatment based on longitudinal cell number data. Treatments include *MT-ND5* mutations, TGFB1 incubation and supplementing uridine, and other_supplements. Other_supple-ments combine 1 mM sodium pyruvate, 1x non-essential amino acids and 1x GlutaMAX. Uridine treatment was supplemented with 50 μg/ml uridine.

Cell line	Assay Day	Heteroplasmy	Treatment	P value

HEK293	12	30 %	*MT-ND5* mutations	0.707
HEK293	18	20 %	*MT-ND5* mutations	0.195
HEK293	23	19 %	*MT-ND5* mutations	0.659
HEK293	26	19 %	*MT-ND5* mutations	0.67
HEK293	30	18 %	*MT-ND5* mutations	0.973
MCF12A	11	11 %	*MT-ND5* mutations	0.961
MCF12A	10	10 %	*MT-ND5* mutations	0.76668
MCF12A	10	10 %	TGFB1	0.00442
MCF12A	12	11 %	Uridine	0.8234
MCF12A	12	11 %	Other_supplements	0.5858
MCF12A	12	11 %	*MT-ND5* mutations	0.9277
HeLa	6	22 %	*MT-ND5* mutations	0.2815

## Appendix A. Appendix

**Table A.2:** Full Enriched KEGG Pathways

pathway	pval	padj	log2err	ES	NES	size	description	

KEGG_RIBOSOME	1.2E-25	1.88E-23	1.311015	−0.83313	−2.80524	83	Ribosome	CP:KEGG
KEGG_OXIDATIVE_PHOSPHORYLATION	4.94E-24	6.5E-22	1.270913	−0.76305	−2.69995	115	Oxidative phosphorylation	CP:KEGG
KEGG_PARKINSONS_DISEASE	9.83E-22	9.63E-20	1.203975	−0.75202	−2.65409	114	Parkinson’s disease	CP:KEGG
KEGG_HUNTINGTONS_DISEASE	2.36E-19	2.07E-17	1.13309	−0.67303	−2.48003	160	Huntington’s disease	CP:KEGG
KEGG_ALZHEIMERS_DISEASE	1.07E-14	6.43E-13	0.986546	−0.65434	−2.3858	142	Alzheimer’s disease	CP:KEGG
KEGG_SPLICEOSOME	5.46E-12	2.17E-10	0.887075	−0.63868	−2.27513	122	Spliceosome	CP:KEGG
KEGG_PROTEASOME	1.21E-08	2.58E-07	0.74774	−0.76307	−2.23254	38	Proteasome	CP:KEGG
KEGG_RNA_POLYMERASE	2.38E-06	2.85E-05	0.627257	−0.75201	−2.08981	29	RNA polymerase	CP:KEGG
KEGG_PYRIMIDINE_METABOLISM	1.84E-07	2.92E-06	0.690132	−0.60819	−2.05718	87	Pyrimidine metabolism	CP:KEGG
KEGG_CARDIAC_MUSCLE_CONTRACTION	8.76E-05	0.000684	0.538434	−0.61517	−1.91487	50	Cardiac muscle contraction	CP:KEGG
KEGG_VIBRIO_CHOLERAE_INFECTION	3.73E-05	0.000321	0.557332	−0.62928	−1.90526	45	*Vibrio cholerae* infection	CP:KEGG
KEGG_CYTOSOLIC_DNA_SENSING_PATHWAY	0.000619	0.003703	0.477271	−0.67518	−1.84442	26	Cytosolic DNA-sensing pathway	CP:KEGG
KEGG_RNA_DEGRADATION	0.000475	0.002936	0.498493	−0.56999	−1.78595	53	RNA degradation	CP:KEGG
KEGG_PATHOGENIC_ESCHERICHIA_COLI_INFECTION	0.000425	0.002674	0.498493	−0.58394	−1.77941	46	Pathogenic *Escherichia coli* infection	CP:KEGG
KEGG_GLUTATHIONE_METABOLISM	0.001526	0.008118	0.45506	−0.61809	−1.77186	34	Glutathione metabolism	CP:KEGG
KEGG_PROTEIN_EXPORT	0.001916	0.009836	0.45506	−0.65377	−1.74102	24	Protein export	CP:KEGG
KEGG_PURINE_METABOLISM	0.000182	0.001283	0.518848	−0.4715	−1.69168	128	Purine metabolism	CP:KEGG
KEGG_PORPHYRIN_AND_CHLOROPHYLL_METABOLISM	0.008516	0.033855	0.38073	−0.63818	−1.65881	20	Porphyrin and chlorophyll metabolism	CP:KEGG
KEGG_BASE_EXCISION_REPAIR	0.005946	0.024931	0.407018	−0.57019	−1.63085	33	Base excision repair	CP:KEGG
KEGG_DNA_REPLICATION	0.013349	0.048705	0.38073	−0.55602	−1.59395	34	DNA replication	CP:KEGG
KEGG_AMINO_SUGAR_AND_NUCLEOTIDE_SUGAR_METABOLISM	0.008921	0.035165	0.38073	−0.53572	−1.58537	40	Amino sugar and nucleotide sugar metabolism	CP:KEGG
KEGG_NUCLEOTIDE_EXCISION_REPAIR	0.007751	0.031228	0.407018	−0.53165	−1.57698	41	Nucleotide excision repair	CP:KEGG
KEGG_PEROXISOME	0.012036	0.045045	0.38073	−0.46359	−1.50635	68	Peroxisome	CP:KEGG
KEGG_AXON_GUIDANCE	0.010106	0.038821	0.38073	0.360163	1.397273	113	Axon guidance	CP:KEGG
KEGG_DILATED_CARDIOMYOPATHY	0.004068	0.018256	0.407018	0.455531	1.577319	57	Dilated cardiomyopathy	CP:KEGG
KEGG_FOCAL_ADHESION	0.000153	0.001118	0.518848	0.402761	1.628305	159	Focal adhesion	CP:KEGG
KEGG_PHOSPHATIDYLINOSITOL_SIGNALING_SYSTEM	0.003516	0.016248	0.431708	0.464431	1.637177	64	Phosphatidylinositol signaling system	CP:KEGG
KEGG_CELL_ADHESION_MOLECULES_CAMS	0.001455	0.007804	0.45506	0.467835	1.676528	68	Cell adhesion molecules (CAMs)	CP:KEGG
KEGG_ABC_TRANSPORTERS	0.003268	0.015317	0.431708	0.575028	1.75762	31	ABC transporters	CP:KEGG
KEGG_HYPERTROPHIC_CARDIOMYOPATHY_HCM	0.00027	0.001792	0.498493	0.536691	1.828867	53	Hypertrophic cardiomyopathy (HCM)	CP:KEGG
KEGG_HEMATOPOIETIC_CELL_LINEAGE	0.001389	0.007532	0.45506	0.628535	1.893618	28	Hematopoietic cell lineage	CP:KEGG
KEGG_ARRHYTHMOGENIC_RIGHT_VENTRICULAR_CARDIOMYOPATHY_ARVC	4.02E-05	0.000343	0.557332	0.564941	1.94953	56	Arrhythmogenic right ventricular cardiomyopathy (ARVC)	CP:KEGG
KEGG_ECM_RECEPTOR_INTERACTION	4.19E-08	7.82E-07	0.719513	0.658765	2.273305	56	ECM-receptor interaction	CP:KEGG

## Appendix B. Supplementary data

Supplementary data to this article can be found online at https://doi.org/10.1016/j.mitoco.2025.03.001.

## References

[1] Warburg O. Über den Stoffwechsel der Carcinomzelle. Naturwissenschaften. 1924;12:1131–1137. 10.1007/BF01504608.

[2] Warburg O. On the origin of cancer cells. Science. 1956;123:309–314. 13298683 10.1126/science.123.3191.309

[3] Weinhouse S. On respiratory impairment in cancer cells. Science (New York, N.Y.). 1956;124:267–269. 10.1126/science.124.3215.267. 13351638

[4] Weinberg F, Hamanaka R, Wheaton WW, Mitochondrial metabolism and ROS generation are essential for Kras-mediated tumorigenicity. Proc Natl Acad Sci USA. 2010;107:8788–8793. 20421486 10.1073/pnas.1003428107 PMC2889315

[5] Tan AS, Baty JW, Dong L-F, Mitochondrial genome acquisition restores respiratory function and tumorigenic potential of cancer cells without mitochondrial DNA. Cell Metab. 2015;21:81–94. 25565207 10.1016/j.cmet.2014.12.003

[6] Viale A, Pettazzoni P, Lyssiotis CA, Oncogene ablation-resistant pancreatic cancer cells depend on mitochondrial function. Nature. 2014;514:628–632. 25119024 10.1038/nature13611 PMC4376130

[7] Luengo A, Li Z, Gui DY, Increased demand for NAD+ relative to ATP drives aerobic glycolysis. Mol Cell. 2021;81:691–707.e6. 10.1016/J.MOLCEL.2020.12.012. 33382985 PMC8315838

[8] Yao C-H, Wang R, Wang Y, Kung C-P, Weber JD, Patti GJ. Mitochondrial fusion supports increased oxidative phosphorylation during cell proliferation. Elife. 2019;8, e41351. 30694178 10.7554/eLife.41351 PMC6351101

[9] Epstein T, Xu L, Gillies RJ, Gatenby RA. Separation of metabolic supply and demand: aerobic glycolysis as a normal physiological response to fluctuating energetic demands in the membrane. Cancer Metabol. 2014;2:1–9. 10.1186/2049-3002-2-7 PMC4060846 24982758

[10] Talmadge JE. Clonal selection of metastasis within the life history of a tumor. Cancer Res. 2007;67:11471–11475. 18089772 10.1158/0008-5472.CAN-07-2496

[11] Greaves M, Maley CC. Clonal evolution in cancer. Nature. 2012;481:306–313. 22258609 10.1038/nature10762 PMC3367003

[12] Klein CA. Selection and adaptation during metastatic cancer progression. Nature. 2013;501:365–372. 24048069 10.1038/nature12628

[13] Ward PS, Patel J, Wise DR, The common feature of leukemia-associated IDH1 and IDH2 mutations is a neomorphic enzyme activity converting alpha-ketoglutarate to 2-hydroxyglutarate. Cancer Cell. 2010;17:225–234. 10.1016/j.ccr.2010.01.020. 20171147 PMC2849316

[14] Pirozzi CJ, Yan H. The implications of IDH mutations for cancer development and therapy. Nat Rev Clin Oncol. 2021;18:645–661. 34131315 10.1038/s41571-021-00521-0

[15] Bardella C, Pollard PJ, Tomlinson I. SDH mutations in cancer. Biochim Biophys Acta Bioenerg. 2011;1807:1432–1443. 10.1016/j.bbabio.2011.07.003 21771581

[16] Valcarcel-Jimenez L, Frezza C. Fumarate hydratase (FH) and cancer: a paradigm of oncometabolism. Br J Cancer. 2023;129:1546–1557. 10.1038/s41416-023-02412-w. 37689804 PMC10645937

[17] Lehtonen R, Kiuru M, Vanharanta S, Biallelic inactivation of fumarate hydratase (FH) occurs in nonsyndromic uterine leiomyomas but is rare in other tumors. Am J Pathol. 2004;164:17–22. 14695314 10.1016/S0002-9440(10)63091-X PMC1602244

[18] Chowdhury R, Yeoh KK, Tian Y-M, The oncometabolite 2-hydroxyglutarate inhibits histone lysine demethylases. EMBO Rep. 2011;12:463–469. 21460794 10.1038/embor.2011.43 PMC3090014

[19] Selak MA, Armour SM, MacKenzie ED, Succinate links TCA cycle dysfunction to oncogenesis by inhibiting HIF-alpha prolyl hydroxylase. Cancer Cell. 2005;7:77–85. 10.1016/j.ccr.2004.11.022. 15652751

[20] Wallace DC. Mitochondria and cancer. Nat Rev Cancer. 2012;12:685–698. 23001348 10.1038/nrc3365 PMC4371788

[21] N S, Rj Y, T F. The mitochondrial basis of aging. Mol Cell. 2016;61. 10.1016/j.molcel.2016.01.028. PMC4779179 26942670

[22] M S-K, Ef F, Dl C, Dm W, Va B. Protecting the mitochondrial powerhouse. Trends Cell Biol. 2015;25. 10.1016/j.tcb.2014.11.002. PMC5576887 25499735

[23] G L, K P, S L, M S-K, N T, Ef F. Mitophagy and neuroprotection. Trends Mol Med. 2020;26. 10.1016/j.molmed.2019.07.002. 31375365

[24] Al-Faze R, Ahmed HA, El-Atawy MA, Mitochondrial dysfunction route as a possible biomarker and therapy target for human cancer. Biomed J. 2025;48,100714. 10.1016/j.bj.2024.100714. 38452973 PMC11743316

[25] Kasahara A, Scorrano L. Mitochondria: from cell death executioners to regulators of cell differentiation. Trends Cell Biol. 2014;24:761–770. 25189346 10.1016/j.tcb.2014.08.005

[26] K KP, A JK, M SP, Regulation of nuclear epigenome by mitochondrial DNA heteroplasmy. Proc Natl Acad Sci USA. 2019;116:16028–16035. 10.1073/pnas.1906896116. 31253706 PMC6689928

[27] Li AM, Ye J. Deciphering the Warburg effect: metabolic reprogramming, epigenetic remodeling, and cell dedifferentiation. Annu Rev Cell Biol. 2024;8:35–58. 10.1146/annurev-cancerbio-062822-120857.

[28] Wallace DC. Mitochondrial genetic medicine. Nat Genet. 2018;50:1642–1649. 10.1038/s41588-018-0264-z. 30374071

[29] Brown WM, George M Jr, Wilson AC. Rapid evolution of animal mitochondrial DNA. Proc Natl Acad Sci USA. 1979;76:1967–1971. 109836 10.1073/pnas.76.4.1967 PMC383514

[30] Alexeyev M, Shokolenko I, Wilson G, LeDoux S. The maintenance of mitochondrial DNA integrity—critical analysis and update. Cold Spring Harbor Perspect Biol. 2013;5, a012641. 10.1101/cshperspect.a012641 PMC3632056 23637283

[31] Ye K, Lu J, Ma F, Keinan A, Gu Z. Extensive pathogenicity of mitochondrial heteroplasmy in healthy human individuals. Proc. Natl. Acad. Sci. U.S.A. 2014;111:10654–10659. 10.1073/pnas.1403521111. arXiv:. 25002485 PMC4115537

[32] Wallace DC. A mitochondrial paradigm of metabolic and degenerative diseases, aging, and cancer: a dawn for evolutionary medicine. Annu Rev Genet. 2005;39:359–407. 16285865 10.1146/annurev.genet.39.110304.095751 PMC2821041

[33] Kim M, Mahmood M, Reznik E, Gammage PA. Mitochondrial DNA is a major source of driver mutations in cancer. Trends in Cancer. 2022;8(1):35–48. 10.1016/j.trecan.2022.08.001 PMC9671861 36041967

[34] Brandon M, Baldi P, Wallace DC. Mitochondrial mutations in cancer. Oncogene. 2006;25:4647–4662. 16892079 10.1038/sj.onc.1209607

[35] Larman TC, DePalma SR, Hadjipanayis AG, Spectrum of somatic mitochondrial mutations in five cancers. Proc Natl Acad Sci USA. 2012;109:14087–14091. 22891333 10.1073/pnas.1211502109 PMC3435197

[36] Stewart JB, Alaei-Mahabadi B, Sabarinathan R, Simultaneous DNA and RNA mapping of somatic mitochondrial mutations across diverse human cancers. PLoS Genet. 2015;11, e1005333. 26125550 10.1371/journal.pgen.1005333 PMC4488357

[37] Hopkins JF, Sabelnykova VY, Weischenfeldt J, Mitochondrial mutations drive prostate cancer aggression. Nat Commun. 2017;8:656. 28939825 10.1038/s41467-017-00377-y PMC5610241

[38] Grandhi S, Bosworth C, Maddox W, Heteroplasmic shifts in tumor mitochondrial genomes reveal tissue-specific signals of relaxed and positive selection. Hum Mol Genet. 2017;26:2912–2922. 10.1093/hmg/ddx172. 28475717 PMC5886292

[39] Yuan Y, Ju YS, Kim Y, Comprehensive molecular characterization of mitochondrial genomes in human cancers. Nat Genet. 2020;52:342–352. 10.1038/s41588-019-0557-x. 32024997 PMC7058535

[40] Kopinski PK, Singh LN, Zhang S, Lott MT, Wallace DC. Mitochondrial DNA variation and cancer. Nat Rev Cancer. 2021;21:431–445. 10.1038/s41568-021-00358-w. 34045735

[41] Scheid AD, Beadnell TC, Welch DR. Roles of mitochondria in the hallmarks of metastasis. Br J Cancer. 2021;124:124–135. 33144695 10.1038/s41416-020-01125-8 PMC7782743

[42] Wallace DC, Bunn CL, Eisenstadt JM. Cytoplasmic transfer of chloramphenicol resistance in human tissue culture cells. J Cell Biol. 1975;67:174–188. 1176530 10.1083/jcb.67.1.174 PMC2109574

[43] Suissa S, Wang Z, Poole J, Ancient mtDNA genetic variants modulate mtDNA transcription and replication. PLoS Genet. 2009;5, e1000474. 19424428 10.1371/journal.pgen.1000474 PMC2673036

[44] Pye D, Kyriakouli DS, Taylor GA, Production of trans mitochondrial cybrids containing naturally occurring pathogenic mtDNA variants. Nucleic Acids Res. 2006;34. e95–e95. 16885236 10.1093/nar/gkl516 PMC1540737

[45] Petros JA, Baumann AK, Ruiz-Pesini E, mtDNA Mutations Increase Tumorigenicity in Prostate Cancer. vol. 102. Proceedings of the National Academy of Sciences; 2005:719–724. 10.1073/pnas.0408894102 PMC545582 15647368

[46] Mok BY, de Moraes MH, Zeng J, A bacterial cytidine deaminase toxin enables CRISPR-free mitochondrial base editing. Nature. 2020;583:631–637. 10.1038/s41586-020-2477-4. 32641830 PMC7381381

[47] Guo X, Xu W, Zhang W, High-frequency and functional mitochondrial DNA mutations at the single-cell level. Proc Natl Acad Sci USA. 2023;120, e2201518120. 36577067 10.1073/pnas.2201518120 PMC9910596

[48] Lei Z, Meng H, Liu L, Mitochondrial base editor induces substantial nuclear off-target mutations. Nature. 2022;606:804–811. 10.1038/s41586-022-04836-5. 35551512

[49] Wei Y, Li Z, Xu K, Mitochondrial base editor DdCBE causes substantial DNA off-target editing in nuclear genome of embryos. Cell Discovery. 2022;8:27. 35304438 10.1038/s41421-022-00391-5 PMC8933521

[50] Sanjana NE, Cong L, Zhou Y, Cunniff MM, Feng G, Zhang F. A transcription activator-like effector toolbox for genome engineering. Nat Protoc. 2012;7:171–192. 10.1038/nprot.2011.431. 22222791 PMC3684555

[51] Kukat A, Kukat C, Brocher J, Generation of *ρ* 0 cells utilizing a mitochondrially targeted restriction endonuclease and comparative analyses. Nucleic Acids Res. 2008;36. e44–e44. 18353857 10.1093/nar/gkn124 PMC2367725

[52] Quinlan CL, Perevoshchikova IV, Hey-Mogensen M, Orr AL, Brand MD. Sites of reactive oxygen species generation by mitochondria oxidizing different substrates. Redox Biol. 2013;1:304–312. 24024165 10.1016/j.redox.2013.04.005 PMC3757699

[53] Nakada K, Sato A, Hayashi J-I. Mitochondrial functional complementation in mitochondrial DNA-based diseases. Int J Biochem Cell Biol. 2009;41:1907–1913. 19464386 10.1016/j.biocel.2009.05.010

[54] Rossignol R, Faustin B, Rocher C, Malgat M, Mazat J-P, Letellier T. Mitochondrial threshold effects. Biochem J. 2003;370:751–762. 12467494 10.1042/BJ20021594 PMC1223225

[55] Stewart JB, Chinnery PF. The dynamics of mitochondrial DNA heteroplasmy: implications for human health and disease. Nat Rev Genet. 2015;16:530–542. 10.1038/nrg3966. 26281784

[56] Criddle DN, Gillies S, Baumgartner-Wilson HK, Menadione-induced reactive oxygen species generation via redox cycling promotes apoptosis of murine pancreatic acinar cells. J Biol Chem. 2006;281:40485–40492. 17088248 10.1074/jbc.M607704200

[57] Gui DY, Sullivan LB, Luengo A, Environment dictates dependence on mitochondrial complex I for NAD+ and aspartate production and determines cancer cell sensitivity to metformin. Cell Metab. 2016;24:716–727. 27746050 10.1016/j.cmet.2016.09.006 PMC5102768

[58] Brown KA, Aakre ME, Gorska AE, Induction by transforming growth factor-*B*1 of epithelial to mesenchymal transition is a rare event in vitro. Breast Cancer Res. 2004;6:1–17. 15084245 10.1186/bcr778 PMC400675

[59] Moses H, Barcellos-Hoff MH. TGF-*β* biology in mammary development and breast cancer. Cold Spring Harbor Perspect Biol. 2011;3, a003277. 10.1101/cshperspect.a003277 PMC3003461 20810549

[60] Zhang Y, Alexander PB, Wang X-F. TGF-*β* family signaling in the control of cell proliferation and survival. Cold Spring Harbor Perspect Biol. 2017;9, a022145. 10.1101/cshperspect.a022145 PMC5378054 27920038

[61] Koczor CA, Shokolenko IN, Boyd AK, Balk SP, Wilson GL, LeDoux SP. Mitochondrial DNA damage initiates a cell cycle arrest by a Chk2-associated mechanism in mammalian cells. J Biol Chem. 2009;284:36191–36201. 19840931 10.1074/jbc.M109.036020 PMC2794735

[62] Mattson MP, Pedersen WA, Duan W, Culmsee C, Camandola S. Cellular and molecular mechanisms underlying perturbed energy metabolism and neuronal degeneration in Alzheimer’s and Parkinson’s diseases. Ann N Y Acad Sci. 1999;893:154–175. 10672236 10.1111/j.1749-6632.1999.tb07824.x

[63] Golpich M, Amini E, Mohamed Z, Azman Ali R, Mohamed Ibrahim N, Ahmadiani A. Mitochondrial dysfunction and biogenesis in neurodegenerative diseases: pathogenesis and treatment. CNS Neurosci Ther. 2017;23:5–22. 27873462 10.1111/cns.12655 PMC6492703

[64] Wang Y, Picard M, Gu Z. Genetic evidence for elevated pathogenicity of mitochondrial DNA heteroplasmy in autism spectrum disorder. PLoS Genet. 2016;12. 10.1371/journal.pgen.1006391. PMC5085253 27792786

[65] Wang Y, Guo X, Hong X, Association of mitochondrial DNA content, heteroplasmies and inter-generational transmission with autism. Nat Commun. 2022;13:3790. 35778412 10.1038/s41467-022-30805-7 PMC9249801

[66] Rowland AA, Voeltz GK. Endoplasmic reticulum–mitochondria contacts: function of the junction. Nat Rev Mol Cell Biol. 2012;13:607–615. 22992592 10.1038/nrm3440 PMC5111635

[67] English AR, Voeltz GK. Endoplasmic reticulum structure and interconnections with other organelles. Cold Spring Harbor Perspect Biol. 2013;5, a013227. 10.1101/cshperspect.a013227 PMC3683900 23545422

[68] Zhong L, Mostoslavsky R. SIRT6: a master epigenetic gatekeeper of glucose metabolism. Transcription. 2010;1:17–21. 21327158 10.4161/trns.1.1.12143 PMC3035182

[69] Cantó C, Menzies KJ, Auwerx J. NA D+ metabolism and the control of energy homeostasis: a balancing act between mitochondria and the nucleus. Cell Metab. 2015;22:31–53. 26118927 10.1016/j.cmet.2015.05.023 PMC4487780

[70] Bertucci F, Finetti P, Birnbaum D. Basal breast cancer: a complex and deadly molecular subtype. Curr Mol Med. 2012;12:96–110. 22082486 10.2174/156652412798376134 PMC3343384

[71] Krug K, Jaehnig EJ, Satpathy S, Proteogenomic landscape of breast cancer tumorigenesis and targeted therapy. Cell. 2020;183:1436–1456. e31. 33212010 10.1016/j.cell.2020.10.036 PMC8077737

[72] Ellis MJ, Gillette M, Carr SA, Connecting genomic alterations to cancer biology with proteomics: the NCI clinical proteomic tumor analysis Consortium. Cancer Discov. 2013;3:1108–1112. 24124232 10.1158/2159-8290.CD-13-0219 PMC3800055

[73] Berger L, Kolben T, Meister S, Expression of H3K4me3 and H3K9ac in breast cancer. J Cancer Res Clin Oncol. 2020;146:2017. 10.1007/S00432-020-03265-Z. 32468423 PMC7324433

[74] Park JS, Sharma LK, Li H, A heteroplasmic, not homoplasmic, mitochondrial DNA mutation promotes tumorigenesis via alteration in reactive oxygen species generation and apoptosis. Hum Mol Genet. 2009;18:1578–1589. 10.1093/hmg/ddp069. 19208652 PMC2733816

[75] Wang Y, Stancliffe E, Fowle-Grider R, Saturation of the mitochondrial NADH shuttles drives aerobic glycolysis in proliferating cells. Mol Cell. 2022;82:3270–3283.e9. 10.1016/j.molcel.2022.07.007. 35973426 PMC10134440

[76] Intlekofer AM, Dematteo RG, Venneti S, Hypoxia induces production of L-2-hydroxyglutarate. Cell Metab. 2015;22:304–311. 10.1016/j.cmet.2015.06.023. 26212717 PMC4527873

[77] Oldham WM, Clish CB, Yang Y, Loscalzo J. Hypoxia-mediated increases in L-2-hydroxyglutarate coordinate the metabolic response to reductive stress. Cell Metab. 2015;22:291–303. 10.1016/j.cmet.2015.06.021. 26212716 PMC4526408

[78] Jiang H, Greathouse RL, Tiche SJ, Mitochondrial uncoupling induces epigenome remodeling and promotes differentiation in neuroblastoma. Cancer Res. 2023;83:181. 10.1158/0008-5472.CAN-22-1029. 36318118 PMC9851961

[79] Moreira JDV, Hamraz M, Abolhassani M, The redox status of cancer cells supports mechanisms behind the Warburg effect. Metabolites. 2016;6:33. 10.3390/metabo6040033. 27706102 PMC5192439

[80] Navas LE, Carnero A. NAD+ metabolism, stemness, the immune response, and cancer. Signal Transduct Targeted Ther. 2021;6:1–20. 10.1038/s41392-020-00354-w. PMC7775471 33384409

[81] Lucena-Cacace A, Otero-Albiol D, Jiménez-García MP, Peinado-Serrano J, Carnero A. NAMPT overexpression induces cancer stemness and defines a novel tumor signature for glioma prognosis. Oncotarget. 2017;8:99514–99530. 10.18632/oncotarget.20577. 29245920 PMC5725111

[82] Ryu KW, Fung TS, Baker DC, Cellular ATP demand creates metabolically distinct subpopulations of mitochondria. Nature. 2024:1–9. 10.1038/s41586-024-08146-w PMC11869630 39506109

[83] Loeb LA. A mutator phenotype in cancer. Cancer Res. 2001;61:3230–3239. 11309271

[84] Ogrunc M, Di Micco R, Liontos M, Oncogene-induced reactive oxygen species fuel hyperproliferation and DNA damage response activation. Cell Death Differ. 2014;21:998–1012. 10.1038/cdd.2014.16. 24583638 PMC4013514

[85] Nieborowska-Skorska M, Kopinski PK, Ray R, Rac2-MRC-cIII–generated ROS cause genomic instability in chronic myeloid leukemia stem cells and primitive progenitors. Blood. 2012;119:4253–4263. 10.1182/blood-2011-10-385658. 22411871 PMC3359741

[86] Hosseinimehr SJ. Flavonoids and genomic instability induced by ionizing radiation. Drug Discov Today. 2010;15:907–918. 10.1016/j.drudis.2010.09.005. 20933097

[87] Piskounova E, Agathocleous M, Murphy MM, Oxidative stress inhibits distant metastasis by human melanoma cells. Nature. 2015;527:186–191. 26466563 10.1038/nature15726 PMC4644103

[88] Zhang H, Ryu D, Wu Y, NAD^+^ repletion improves mitochondrial and stem cell function and enhances life span in mice. Science. 2016;352:1436–1443. 10.1126/science.aaf2693. 27127236

[89] Amjad S, Nisar S, Bhat AA, Role of NAD+ in regulating cellular and metabolic signaling pathways. Mol Metabol. 2021;49, 101195. 10.1016/j.molmet.2021.101195. PMC7973386 33609766

[90] Xie N, Zhang L, Gao W, NAD+ metabolism: pathophysiologic mechanisms and therapeutic potential. Signal Transduct Targeted Ther. 2020;5:1–37. 10.1038/s41392-020-00311-7. PMC7539288 33028824

[91] Jiang Y, Luo Z, Gong Y, Fu Y, Luo Y. NAD+ supplementation limits triple-negative breast cancer metastasis via SIRT1-P66Shc signaling. Oncogene. 2023;42:808–824. 10.1038/s41388-023-02592-y. 36690678

[92] Pang N, Hu Q, Zhou Y, Nicotinamide adenine dinucleotide precursor suppresses hepatocellular cancer progression in mice. Nutrients. 2023;15:1447. 36986177 10.3390/nu15061447 PMC10055624

[93] Ikeda H, Kawase K, Nishi T, Immune evasion through mitochondrial transfer in the tumour microenvironment. Nature. 2025;638:225–236. 10.1038/s41586-024-08439-0. 39843734 PMC11798832

[94] Saha T, Dash C, Jayabalan R, Intercellular nanotubes mediate mitochondrial trafficking between cancer and immune cells. Nat Nanotechnol. 2022;17:98–106. 10.1038/s41565-021-01000-4. 34795441 PMC10071558

[95] Baldwin JG, Gattinoni L. Cancer cells hijack T-cell mitochondria. Nat Nanotechnol. 2022;17:3–4. 34795442 10.1038/s41565-021-01006-y

[96] Sanjana NE, Cong L, Zhou Y, Cunniff MM, Feng G, Zhang F. A transcription activator-like effector toolbox for genome engineering. Nat Protoc. 2012;7:171–192. 10.1038/nprot.2011.431. 22222791 PMC3684555

[97] Borowicz S, Van Scoyk M, Avasarala S, The Soft Agar Colony Formation Assay. 2014. 10.3791/51998. PMC4353381 25408172

[98] Chaudhry A, Shi R, Luciani DS. A pipeline for multidimensional confocal analysis of mitochondrial morphology, function, and dynamics in pancreatic *β*-cells. Am J Physiol Endocrinol Metabol. 2020;318:E87–E101. 10.1152/ajpendo.00457.2019. PMC7052579 31846372

[99] Spinazzi M, Casarin A, Pertegato V, Salviati L, Angelini C. Assessment of mitochondrial respiratory chain enzymatic activities on tissues and cultured cells. Nat Protoc. 2012;6(7):1235–1246. 10.1038/nprot.2012.058, 2012 7. 22653162

[100] Rodríguez-Nuevo A, Torres-Sanchez A, Duran JM, De Guirior C, Martínez-Zamora MA, Böke E. Oocytes maintain ROS-free mitochondrial metabolism by suppressing complex I. Nature. 2022;7920(607):756–761. 10.1038/s41586-022-04979-5, 2022 607. PMC9329100 35859172

[101] Krueger F. TrimGalore, Babraham Bioinformatics. Babraham Institute; 2019.

[102] Martin M. Cutadapt removes adapter sequences from high-throughput sequencing reads. EMBnet.journal. 2011;17(1). 10.14806/ej.17.1.200. Next Generation Sequencing Data AnalysisDO.

[103] Andrews S. FastQC: A Quality Control Tool for High Throughput Sequence Data. 2010.

[104] Dobin A, Davis CA, Schlesinger F, STAR: ultrafast universal RNA-seq aligner. Bioinformatics. 2013;29:15–21. 10.1093/bioinformatics/bts635. 23104886 PMC3530905

[105] Robinson MD, McCarthy DJ, Smyth GK. edgeR: a Bioconductor package for differential expression analysis of digital gene expression data. Bioinformatics. 2010;26:139–140. 10.1093/bioinformatics/btp616. 19910308 PMC2796818

[106] Chen EY, Tan CM, Kou Y, Enrichr: interactive and collaborative HTML5 gene list enrichment analysis tool. BMC Bioinf. 2013;14:1–14. 10.1186/1471-2105-14-128 PMC3637064 23586463

[107] Kuleshov MV, Jones MR, Rouillard AD, Enrichr: a comprehensive gene set enrichment analysis web server 2016 update. Nucleic Acids Res. 2016;44:W90–W97. 27141961 10.1093/nar/gkw377 PMC4987924

[108] Xie Z, Bailey A, Kuleshov MV, Gene set knowledge discovery with Enrichr. Current protocols. 2021;1:e90. 33780170 10.1002/cpz1.90 PMC8152575

[109] Shannon P, Markiel A, Ozier O, Cytoscape: a software environment for integrated models of biomolecular interaction networks. Genome Res. 2003;13:2498–2504. 14597658 10.1101/gr.1239303 PMC403769

