# Supplementary material for: Exploring the oncogenic impact of heteroplasmic de novo MT-ND5 truncating mutations
Source: Mitochondrial Commun. Author manuscript; Available in PMC 2025 Jul 16. (PMC12266708; doi:10.1016/j.mitoco.2025.03.001)
Supplement: Figure Supplement 1 [file NIHMS2077304-supplement-Figure_Supplement_1.pdf]

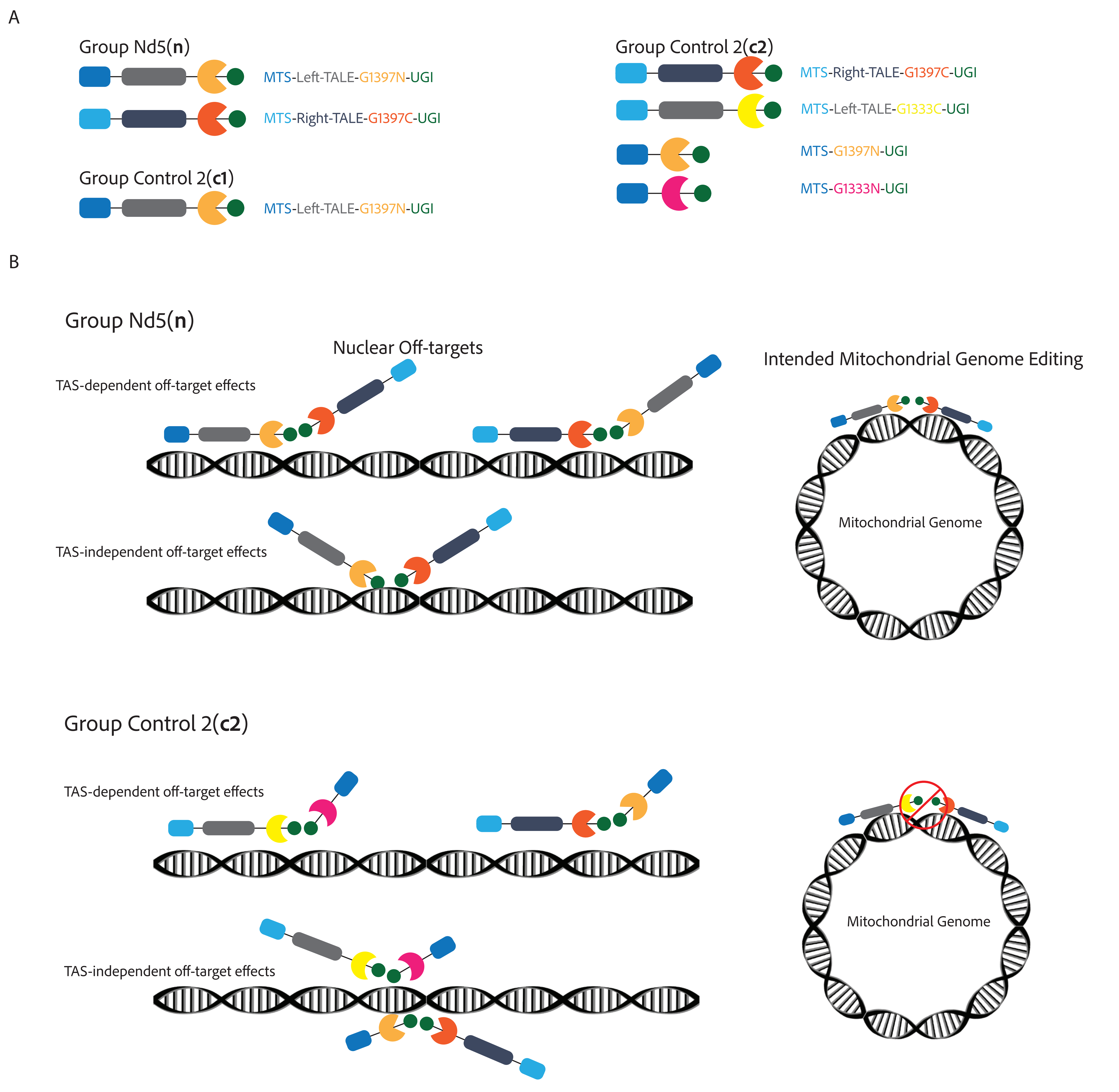
A
Group Nd5(**n**)
MTS-Left-TALE-G1397N-UGI
MTS-Right-TALE-G1397C-UGI
Group Control 2(**c1**)
MTS-Left-TALE-G1397N-UGI
Group Control 2(**c2**)
MTS-Right-TALE-G1397C-UGI
MTS-Left-TALE-G1333C-UGI
MTS-G1397N-UGI
MTS-G1333N-UGI
B
Group Nd5(**n**)
Nuclear Off-targets
TAS-dependent off-target effects
TAS-independent off-target effects
Intended Mitochondrial Genome Editing
Mitochondrial Genome
Group Control 2(**c2**)
TAS-dependent off-target effects
TAS-independent off-target effects
Mitochondrial Genome
