# Supplementary material for: Exploring the oncogenic impact of heteroplasmic de novo MT-ND5 truncating mutations
Source: Mitochondrial Commun. Author manuscript; Available in PMC 2025 Jul 16. (PMC12266708; doi:10.1016/j.mitoco.2025.03.001)
Supplement: Supplement_2_plasmid_sequence [file NIHMS2077304-supplement-Supplement_2_plasmid_sequence.pdf]

MTS-Left-TALE-G1397N-UGI plasmid has an architecture of (from N- to C-terminus): SOD2 MTS-3xHA-leftTALE-2aa linker-G1397N (DddAtox left half) -4aa linker-1x-UGI– SOD2 3’UTR

MTS-Right-TALE-G1397C-UGI plasmid has an architecture of (from N- to C-terminus):
COX8A MTS–3xFLAG-rightTALE-2aa linker-G1397C (DddAtox right half) -4aa linker-1x-UGI-ATP5B 3'UTR

MTS-Left-TALE-G1333C-UGI plasmid has an architecture of (from N- to C-terminus):
COX8A MTS–3xFLAG-leftTALE-2aa linker-G1333C (DddAtox right half) -4aa linker-1x-UGI-ATP5B 3'UTR

MTS-G1397N-UGI plasmid has an architecture of (from N- to C-terminus):
SOD2 MTS-3xHA-2aa linker-G1397N (DddAtox left half) -4aa linker-1x-UGI– SOD2 3’UTR

MTS-G1333N-UGI plasmid has an architecture of (from N- to C-terminus):
SOD2 MTS-3xHA-2aa linker-G1333N (DddAtox left half) -4aa linker-1x-UGI– SOD2 3’UTR

TALE array sequence (TAS) domains are annotated as: bold for N-terminal domain, underlined for RVD and bolded italics for C-terminal domain.

Left TALE:
M**DIADLRTLGYSQQQQEKIKPKVRSTVAQHHEALVGHGFTHAHIVALSQHPAALGTVAVKYQDMIAALPEATHEAIVGVGKQWSGARALEALLTVAGELRGPPLQLDTGQLLKIAKRGGVTAVEAVHAWRNALTGAPLN**LTPEQVVAIASNGGGKQALETVQRLLPVLCQAHGLTPEQVVAIASNNGGKQALETVQRLLPVLCQAHGLTPEQVVAIASNNGGKQALETVQRLLPVLCQAHGLTPEQVVAIASHDGGKQALETVQRLLPVLCQAHGLTPEQVVAIASNIGGKQALETVQRLLPVLCQAHGLTPEQVVAIASNGGGKQALETVQRLLPVLCQAHGLTPEQVVAIASHDGGKQALETVQRLLPVLCQAHGLTPEQVVAIASNGGGKQALETVQRLLPVLCQAHGLTPEQVVAIASNNGGKQALETVQRLLPVLCQAHGLTPEQVVAIASHDGGKQALETVQRLLPVLCQAHGLTPEQVVAIASNGGGKQALETVQRLLPVLCQAHGLTPEQVVAIASHDGGRPALE**SIVAQLSRPDPALAALTNDHLVALACLGGRPALDAVKKGLG**

Right TALE:
M**DIADLRTLGYSQQQQEKIKPKVRSTVAQHHEALVGHGFTHAHIVALSQHPAALGTVAVKYQDMIAALPEATHEAIVGVGKQWSGARALEALLTVAGELRGPPLQLDTGQLLKIAKRGGVTAVEAVHAWRNALTGAPLN**LTPEQVVAIASNNGGKQALETVQRLLPVLCQAHGLTPEQVVAIASNNGGKQALETVQRLLPVLCQAHGLTPEQVVAIASHDGGKQALETVQRLLPVLCQAHGLTPEQVVAIASNIGGKQALETVQRLLPVLCQAHGLTPEQVVAIASNGGGKQALETVQRLLPVLCQAHGLTPEQVVAIASHDGGKQALETVQRLLPVLCQAHGLTPEQVVAIASNGGGKQALETVQRLLPVLCQAHGLTPEQVVAIASNNGGKQALETVQRLLPVLCQAHGLTPEQVVAIASHDGGKQALETVQRLLPVLCQAHGLTPEQVVAIASNGGGKQALETVQRLLPVLCQAHGLTPEQVVAIASHDGGKQALETVQRLLPVLCQAHGLTPEQVVAIASNNGGKQALE**TVQRLLPVLCQAHGLTPEQVVAIASNNGGRPALESIVAQLSRPDPALAALTNDHLVALACLGGRPALDAVKKGLG**

COX8A-MTS:
SVLTPLLLRGLTGSARRLPVPRAKIHSL

SOD2-MTS:
LSRAVCGTSRQLAPVLGYLGSRQKHSLPD

3xFLAG:
DYKDHDGDYKDHDIDYKDDDDK

3xHA:
YPYDVPDYAGYPYDVPDYAGYPYDVPDYA

2aa linker: GS

4aa linker: SGGS

ATP5B 3'UTR:
ACCACGATCGTTATGCTGATCATACCCTAATGATCCCAGCAAGATAATGTCCTGTCTTCTAAGATGTGCATCAAGCC
TGGTACATACTGAAAACCCTATAAGGTCCTGGATAATTTTTGTTTGATTATTCATTGAAGAAACATTTATTTTCCAAT
TGTGTGAAGTTTTTGACTGTTAATAAAAGAATCTGTCAACCATCAAAAAAAAAAAAAAA

SOD2 3'UTR:
ACCACGATCGTTATGCTGATCATACCCTAATGATCCCAGCAAGATAATGTCCTGTCTTCTAAGATGTGCATCAAGCC
TGGTACATACTGAAAACCCTATAAGGTCCTGGATAATTTTTGTTTGATTATTCATTGAAGAAACATTTATTTTCCAAT
TGTGTGAAGTTTTTGACTGTTAATAAAAGAATCTGTCAACCATCAAAAAAAAAAAAAAA

G1397N (DddAtox left half):
GSYALGPYQISAPQLPAYNGQTVGTFYYVNDAGGLESKVFSSGGPTPYPNYANAGHVEGQSALFMRDNGISEGLVFHN
NPEGTCGFCVNMTETLLPENAKMTVVPPEG

G1397C (DddAtox right half):
AIPVKRGATGETKVFTGNSNSPKSPTKGGC

G1333N (DddAtox left half):
GSYALGPYQISAPQLPAYNGQTVGTFYYVNDAGGLESKVFSSGG

G1333C (DddAtox right half):
PTPYPNYANAGHVEGQSALFMRDNGISEGLVFHNNPEGTCGFCVNMTETLLPENAKMTVVPPEGAIPVKRGATGETK
VFTGNSNSPKSPTKGGC
